# *TP53 *mutation status and gene expression profiles are powerful prognostic markers of breast cancer

**DOI:** 10.1186/bcr1675

**Published:** 2007-05-15

**Authors:** Anita Langerød, Hongjuan Zhao, Ørnulf Borgan, Jahn M Nesland, Ida RK Bukholm, Tone Ikdahl, Rolf Kåresen, Anne-Lise Børresen-Dale, Stefanie S Jeffrey

**Affiliations:** 1 Department of Genetics, Institute for Cancer Research, Rikshospitalet-Radiumhospitalet Medical Center, Oslo, Norway N-0310; 2 Department of Surgery, Stanford University School of Medicine, Stanford, CA 94305, USA; 3 Department of Urology, Stanford University School of Medicine, Stanford, CA 94305, USA; 4 Department of Mathematics, University of Oslo, Oslo, Norway N-0316; 5 Department of Pathology, Rikshospitalet-Radiumhospitalet Medical Center, Oslo, Norway N-0310; 6 Faculty of Medicine, University of Oslo, Oslo, Norway; 7 Department of Surgery, Akershus University Hospital, Nordbyhagen, Norway N-1474; 8 Cancer Center, Ullevål University Hospital, Oslo, Norway N-0407; 9 Department of Surgery, Ullevål University Hospital, Oslo, Norway N-0407

## Abstract

**Introduction:**

Gene expression profiling of breast carcinomas has increased our understanding of the heterogeneous biology of this disease and promises to impact clinical care. The aim of this study was to evaluate the prognostic value of gene expression-based classification along with established prognostic markers and mutation status of the *TP53 *gene (tumour protein p53) in a group of breast cancer patients with long-term (12 to 16 years) follow-up.

**Methods:**

The clinical and histopathological parameters of 200 breast cancer patients were studied for their effects on clinical outcome using univariate/multivariate Cox regression. The prognostic impact of mutations in the *TP53 *gene, identified using temporal temperature gradient gel electrophoresis and sequencing, was also evaluated. Eighty of the samples were analyzed for gene expression using 42 K cDNA microarrays and the patients were assigned to five previously defined molecular expression groups. The strength of the gene expression based classification versus standard markers was evaluated by adding this variable to the Cox regression model used to analyze all samples.

**Results:**

Both univariate and multivariate analysis showed that *TP53 *mutation status, tumor size and lymph node status were the strongest predictors of breast cancer survival for the whole group of patients. Analyses of the patients with gene expression data showed that *TP53 *mutation status, gene expression based classification, tumor size and lymph node status were significant predictors of survival. Breast cancer cases in the 'basal-like' and 'ERBB2^+^' gene expression subgroups had a very high mortality the first two years, while the 'highly proliferating luminal' cases developed the disease more slowly, showing highest mortality after 5 to 8 years.  The *TP53 *mutation status showed strong association with the 'basal-like' and 'ERBB2^+^' subgroups, and tumors with mutation had a characteristic gene expression pattern.

**Conclusion:**

*TP53 *mutation status and gene-expression based groups are important survival markers of breast cancer, and these molecular markers may provide prognostic information that complements clinical variables. The study adds experience and knowledge to an ongoing characterization and classification of the disease.

## Introduction

The evidence suggesting that molecular profiling can refine breast cancer prognosis are so far promising. From cDNA microarray analysis of locally advanced breast carcinomas, Perou and colleagues [1] identified five subgroups based on their distinct gene expression patterns. The subgroups were shown to differ with respect to outcome [2], and have also been identified in other datasets [3]. van't Veer and colleagues [4] analyzed node-negative breast cancer patients under the age of 55 years using DNA microarrays and identified a 'poor prognosis signature' that predicted short interval to distant metastasis. A larger set of samples was studied by van de Vijver and colleagues [5] to confirm the predictive value of this signature in women under 53 years of age. Other datasets have been analyzed with similar findings of molecular subgroups with different clinical outcomes [6-10]. However, there are few published studies with a relatively large number of patients with long-term follow-up.

Several well-established clinical, histopathological and molecular factors are today used as prognostic and predictive markers of breast cancer. These include patient age, tumor size, lymph node status, presence of distant metastasis (TNM-stage; tumor, node, metastasis), histological type, tumor grade, and estrogen receptor (ER), progesterone receptor (PR) and ERBB2/HER-2 status. Improvements of prognostic criteria have been achieved by optimally combining available markers. The National Cancer Institute [11] and St Gallen Conference [12] provide adjuvant treatment guidelines based on these markers. Currently, TNM-staging [13], the Nottingham Prognostic Index [14] and Adjuvant Online [15] are the most commonly used integrated prognostic models.

*TP53 *mutation status is rarely obtained for routine analysis, despite accumulating evidence of its prognostic value. Mutations in the *TP53 *gene have been reported to be present in more than half of all cancer cases [16]; however, the frequency shows variation between types/subtypes of cancer. In breast cancer, the frequency of *TP53 *gene mutations is approximately 20% to 30%. Acquiring a *TP53 *mutation has been suggested to be an early event in breast cancer development and it is related to poor prognosis and chemo resistance [17]. Allelic imbalance (AI) (or loss of heterozygosity (LOH)) at chromosome location 17p13, where the *TP53 *gene is located, has been reported in more than half of breast carcinomas [18]. Traditionally, AI is considered as an additional event eliminating the *TP53 *tumor suppressor function.

In this study we address the question of whether gene expression profiles offer better prognostic information in patients with long-term follow-up. We performed univariate and multivariate analysis of seven standard markers and *TP53 *mutation status for the total group of breast cancer patients. We then analyzed a large subset of these tumors using cDNA microarrays and assigned the samples to five previously defined molecular expression groups. The strength of gene expression-based classification versus standard markers was evaluated by adding this variable to the Cox regression model used to analyze all samples. This is the first report that includes both gene expression groups and *TP53 *mutation status in a multivariate analysis.

## Materials and methods

### Patient material

A series of 212 primary breast cancer cases were studied; 80 of these tumors were analyzed using cDNA microarrays, along with one normal breast tissue sample collected from breast reduction surgery. Patient samples were sequentially collected at Ullevål University Hospital from 1990 to 1994 (IRB approval 350, protocol 75026). The last update of patient information was done in 2006, providing an observation time of 12 to 16 years. Patients were followed until death or emigration, and only 12 patients were lost to follow-up. The average age of the 80 cases analyzed by cDNA microarrays was 65.0 years at time of primary surgery (range 28.2 to 87.7 years), similar to the average age of 64.4 years (range 28.2 to 91.5 years) for the total series. The 80 cases were selected from the total series based only on sufficient amount of fresh frozen tissue for microarray analysis. Consequently, a slightly higher fraction of patients with larger tumor size was observed in this subcohort. A summary of the clinical and histopathological data of the patients is shown in Table 1 (see Additional file 1 for more detailed information). All patients were treated according to Norwegian national guidelines at the time of diagnosis [19]. Patients receiving adjuvant systemic therapy were given nine courses of CMF (cyclophosphamide, methotrexate, 5-fluorouracil) and/or Tamoxifen for two years. Dosage of radiation given as adjuvant treatment was dependent on indication; after breast conserving therapy the mammary gland was given 50 Gy (2 Gy × 25). The number of samples entered into the survival analyses is smaller than 212 (full dataset) and 80 (subset with gene expression data); excluding patients with missing information or distant metastases at the time of diagnosis and primary surgery, leaves us with a maximum number of 200 (full set) and 77 (subset) patients.

**Table 1:** Clinical and histopathological characteristics of cases included in the study

	All cases (*n *= 212)		Microarray (*n *= 80)	
		
Characteristics	Number	Percent	Number	Percent
Age (year)				
<45	24	11.3	6	7.5
45–55	37	17.5	12	15.0
55–65	38	17.9	20	25.0
65–75	50	23.6	21	26.3
≥75	63	29.7	21	26.3
Gender				
Female	211	99.5	79	98.8
Male	1	0.5	1	1.2
Type				
Ductal	132	64.4	51	63.8
Lobular	53	25.8	22	27.5
Mucinous	8	3.9	3	3.8
Medullary	4	2.0	0	0
DCIS with microinvasion	1	0.5	1	1.3
Tubulolobular	2	1.0	2	2.5
Other	5	2.4	1^a^	1.3
Unknown	7		0	
Tumor size				
pT1 (≤2 cm)	71	34.6	20	26.0
pT2 (>2 ≤5 cm)	112	54.6	44	57.1
pT3 (>5 cm)	15	7.3	8	10.4
pT4 (infiltrating growth)	7	3.4	5	6.5
Unknown	7		3	
Node status				
pN0 (negative)	95	44.8	33	41.3
pN1 (1–3 positive)	48	22.6	23	28.8
pN2–N3 (≥4 positive)	33	15.6	12	15.0
pNX (Nodes not removed)	36	17.0	12	15.0
Grade				
1	19	9.0	6	7.5
2	141	66.5	53	66.3
3	48	22.6	21	26.3
Unknown	4		0	
Estrogen receptor				
Positive	109	57.7	45	62.5
Negative	80	42.3	27	37.5
Unknown	23		8	
Progesteron receptor				
Positive	130	62.8	51	67.1
Negative	77	37.2	25	32.9
Unknown	5		4	
*TP53 *mutation				
Mutation	48	23.6	20	25.0
Wild type	155	76.4	60	75.0
Unknown	9		0	
Gene expression group				
Luminal A			23	28.8
Highly proliferating luminal			15	18.8
Normal-like			20	25.0
Basal-like			12	15.0
ERBB2+			10	12.5
Adjuvant therapy				
Radiation therapy				
Yes	60	31.1	25	32.9
No	133	68.9	51	67.1
Unknown	19		4	
Chemotherapy^b^				
Yes	27	12.9	11	14.1
No	182	87.1	67	85.9
Unknown	3		2	
Hormonal treatment^c^				
Yes	53	25.1	25	31.6
No	158	74.9	54	68.4
Unknown	1			
Recurrence (distant metastisis)				
Positive	72	34.1	34	42.5
Negative	139	65.9	46	57.5
Unknown	1		0	
Status				
Alive	75	37.5	25	32.5
Dead of disease	63	31.5	30	39.0
Dead of other cause	60	30.0	20	26.0
Emmigrated	2	1.0	2	2.6
Unknown	12		3	

^a^Rare type with a metaplastic core and spindle cell component. ^b^CMF × 9 (cyclophosphamide, methotrexate, 5-fluorouracil). ^c^Tamoxifen; 2 years. DCIS, ductal carcinoma *in situ*.

### Tissue acquisition and histology evaluation

Primary breast carcinoma tissue was snap frozen and stored at -80°C. Frozen sections stained with hematoxylin/eosin were reviewed to confirm tumor content, and specimens in which at least 5% of the cells were carcinoma cells were included in this study. The majority of samples (80%) analyzed using microarrays had at least 40% tumor cell content. Sections from paraffin embedded tissue were re-evaluated by an experienced breast pathologist (JMN) to classify and grade the carcinomas according to the modified Scarff-Bloom-Richardson method [20] (Table 1).

### *TP53 *and hormone-receptor analysis

DNA was isolated from both peripheral blood cells (leukocytes) and tumor tissue using chloroform/phenol extraction followed by ethanol precipitation (Nuclear Acid Extractor 340A; Applied Biosystems, Foster City, California, USA) according to standard procedures. *TP53 *mutation detection in tumor DNA was performed by prescreening exon 2–11 using temporal temperature gradient gel electrophoresis (TTGE) [21]. Samples with aberrant migrating bands from TTGE were sequenced (ABI PRISM™ 377 DNA Sequencer, Applied Biosystems) to determine the nature of the mutation. AI analysis was performed using the ABI 310 capillary electrophoresis on two different highly polymorphic markers in the *TP53 *locus, one located in intron 1 [22] and the other downstream of exon 11 [23]. AI was scored according to a threshold of 0.84 (ratio between two allele variants in tumor divided by ratio between two alleles in blood) [24]. At least one of the polymorphic markers had to show AI to score positive. The ER and PR were analyzed using both immunohistochemistry (IHC) and biochemical/ligand-binding assay (Abbott Diagnostics, Abbott Park, Illinois, USA). The results from IHC were used in our data analysis since that is the current recommended method; however, in a few cases where IHC had not been performed, results from a biochemical/ligand binding assay were used.

### Microarray experiments and hierarchical clustering

**Figure 1:**
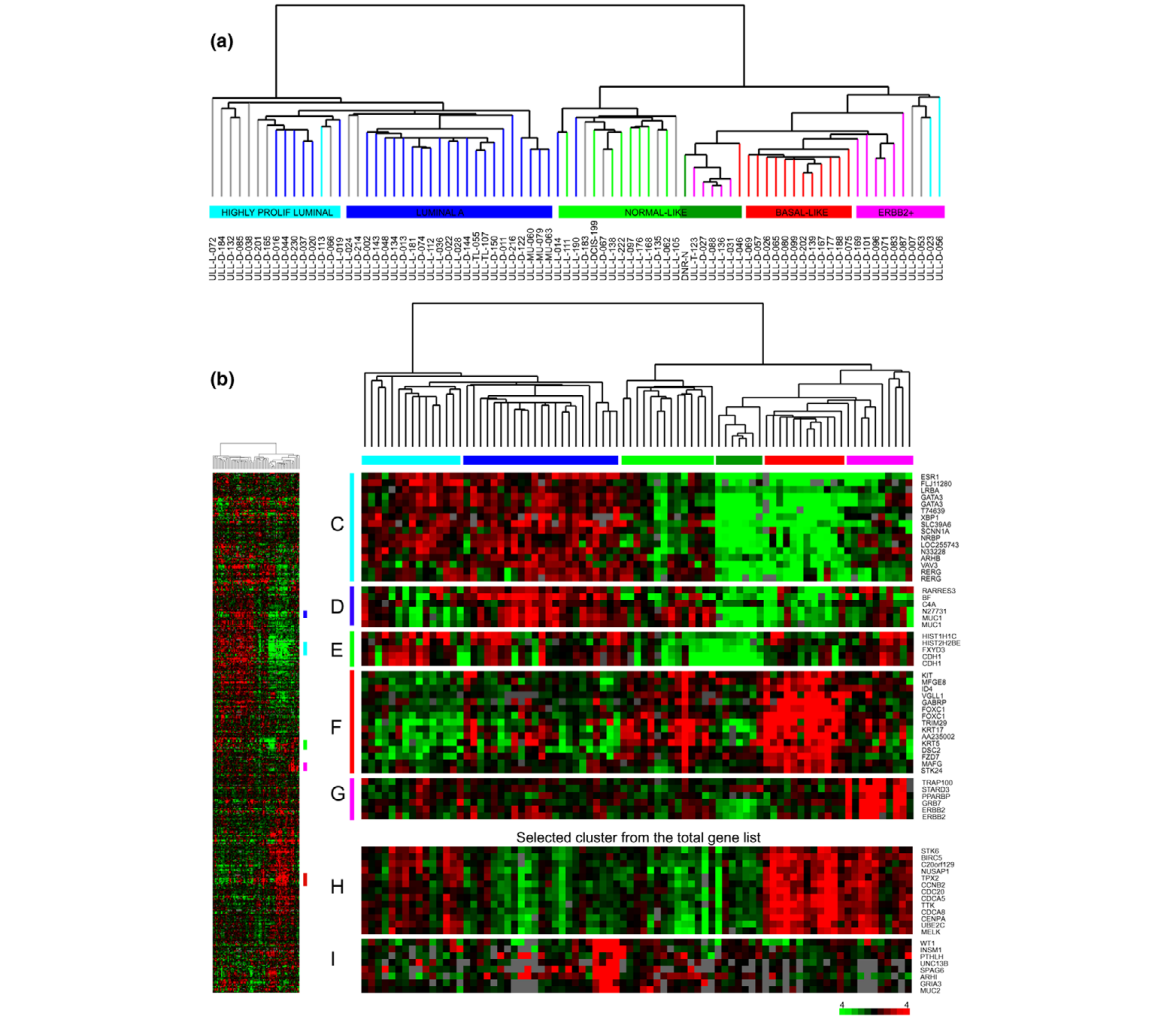
Hierarchical clustering using 'intrinsic' genes. **(a) **Five groups were identified, namely the highly proliferating luminal (light blue), luminal A (dark blue), normal-like (green), basal-like (red) and ERBB2^+ ^(magenta) groups, which were used in the survival analysis. **(b) **Hierarchical clustering was performed using 540 clones, representing 496 unique genes from the 'intrinsic' gene list. The individual color of the dendrogram branches reflects the correlation with centroids previously described, and tumors with low correlation (<0.2) with a specific subtype are here indicated by gray branches. Gene clusters characterizing the five groups (a) involve genes encoding, for example, **(c) **estrogen receptor (ER), **(d) **MUC1, **(e) **cadherin 1 (E-cadherin; CDH1), **(f) **FOXC1 and **(g) **ERBB2. Since very few genes associated with cell division and proliferation are part of the 'intrinsic' gene list, such a cluster was selected from the total list of genes (Additional file 4), clustered and organized according to **(h) **the 'intrinsic' dendrogram to show the difference in proliferation between the two luminal groups. **(i) **In the same manner, a gene cluster characteristic of the mucinous breast carcinomas was made from the total list of genes.

Total RNA was isolated from snap frozen tumor tissue using TRIzol^® ^solution (Invitrogen™, Carlsbad, California, USA). The concentration of total RNA was determined using an HP 8453 spectrophotometer (Hewlett Packard) and the integrity of the RNA was assessed using a 2100 Bioanalyzer (Agilent, Santa Clara, California, USA). Linear amplification of total RNA was performed using an optimized protocol previously described [25]. Amplified tumor RNA was labeled by Cy5 and amplified RNA from Universal Human Reference total RNA (Stratagene^®^, La Jolla, California, USA) was labeled by Cy3. The labeling and hybridization of amplified RNA to cDNA microarrays, containing more than 42,000 elements, was performed as previously described [25]. Experimental protocols can be found at the referred web site [26]. Images of the arrays was acquired using a Gene Pix 4000B scanner (Axon Instruments, Sunnyvale, California, USA), and analyzed using Gene Pix Pro 3.0/4.0/4.1 software and by visual inspection. Data were entered in the Stanford Microarray Database [27], and intensity levels were normalized. Data were filtered for spot quality and included in the analysis if the pixels within a spot showed a regression correlation of at least 0.6 or if the signal intensity of both sample and reference were at least 1.5 over background. A hierarchical clustering algorithm integrated into the Stanford Microarray Database was applied to group genes and samples on the basis of their similarities in expression, and the results were visualized using TreeView [28] and Java TreeView [29] software (for analysis software links, see [30]). Prior to clustering analysis, the data were centered to median expression of each gene across the dataset. The hierarchical clustering shown in Figure 1 was performed using previously described 'intrinsic' genes [3] consisting of 540 clones (representing 496 genes corresponding to a single unique UniGene cluster) whose expression was measurable in at least 70% of the samples. The 'intrinsic' set of genes consisted of genes with significantly greater variation in expression between different tumors than between paired samples from the same tumor, the name reflecting that genes were selected to optimally identify intrinsic characteristics of breast tumors.

### Survival analysis

In the analysis of breast cancer death for all patients and for patients with gene expression data, we used the Kaplan-Meier estimator and univariate Cox regression to assess the marginal effect of each factor (that is, when not correcting for the effect of other factors). The joint effect of two or more factors was assessed using multivariate Cox regression. A parsimonious Cox regression model with only significant factors was obtained by backwards elimination starting with all factors, and the final model was checked for all possible two-factor interactions. The *P *value for the total effect of a factor was calculated from the (partial) likelihood ratio statistic, while the Wald test statistic was used to compute the *P *value for each level of a multilevel factor. The proportional hazards assumption of Cox regression was checked using the test of Grambsch and Therneau [31] with Kaplan-Meier weights as implemented in S-PLUS (version 6.1 for Windows. Insightful Corporation, Seattle, Washington, USA).

### Additional statistical analysis

Pearson correlation analysis was used (Microsoft Excel 2000) to find the correlation between the gene expression profile of a single sample and five previously defined centroids [3]. The five centroids were based on a set of tumor samples from advanced breast cancer cases and represent the average expression profiles of sample subgroups defined by hierarchical clustering using genes that showed more similar expression between two samples from the same tumor than between any other tumor ('intrinsic' genes). Cross-tabulation and Pearson *X*^2^-test or Fishers Exact test (when appropriate) were performed using SPSS (version 13.0. SPSS Inc, Chicago, Illinois, USA) when studying distribution of clinical, histopathological or molecular genetic parameters. Genes with potential significant changes in expression between tumors having *TP53 *mutations and tumors with wild-type *TP53 *were identified using the significance analysis of microarray (SAM) procedure [32,33]. Data from 27,393 clones whose expression was measurable in at least 80% of the samples were included in the analysis.

## Results

### Gene expression based classification

**Figure 2:**
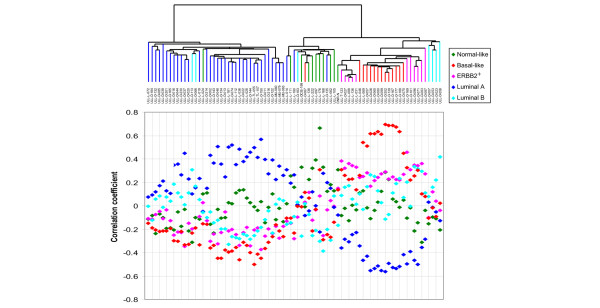
Correlation with five centroids. The expression profiles of the samples were correlated with five previously defined centroids (listed with color codes). The correlation coefficients were plotted according to the dendrogram in Figure 1 and a continuous and opposite curve-pattern of luminal A versus basal-like is evident. The subcluster of luminal samples with the second highest correlation with the luminal B centroid is named 'highly proliferating luminals' in our study.

The 80 breast tumor samples were assigned to five different subgroups according to their gene expression pattern; luminal A, highly proliferating luminals, normal-like, basal-like and ERBB2^+^. The assignment of tumors into subgroups was based on hierarchical clustering using the 540 previously identified 'intrinsic' genes. The resulting dendrogram showed two main groups of breast tumors (Figure 1); those with high expression of the ER-related gene cluster and luminal type characteristics (left branch), and those showing a lower relative expression of the ER-related cluster (right branch). Further subdivision of the samples identified five groups similar to those previously described [2] also in this set of breast tumors. The correlation between the gene expression profile of each sample and five previously described centroids [2] were calculated and a scatter chart was made (Figure 2) according to the order of samples in the dendrogram from hierarchical clustering (Figure 1). The correlation of each sample to each of the centroids showed a continuous wave pattern over the sample set, and visualized how each sample carries elements from different profiles. The luminal A and basal-like breast tumors showed a pronounced opposite proportional pattern.

In the subsequent Cox regression analysis the breast tumor samples were assigned to five subgroups based on hierarchical clustering, combined with the pattern of centroid correlation and fine-tuned by gene expression pattern of characteristic subgroup markers. It should be emphasized that the classification method is unsupervised, meaning that the samples are grouped solely based on the gene expression data.

### Survival analysis of all patients

In Table 2 (Univariate analysis) relative risks (hazard ratios) with 95% confidence intervals (CI) of univariate Cox regressions for all factors considered are shown. *P *values for testing the hypothesis of no marginal effect of a factor and its levels are also given. Tumor size and *TP53 *status are the two most significant factors, but lymph node status also has a significant effect. Patients with *TP53 *mutations have a breast cancer death rate that is four to five times higher than for those without mutations. Patients with tumor size T3–T4 have a breast cancer death rate that is about four times higher than for those with tumor size T1, while patients with tumor size T2 have a breast cancer death rate that is about double that of those with size T1. Patients with four or more positive lymph nodes have a breast cancer death rate that is about three times higher than for those with negative lymph nodes. The effect of *TP53 *status and tumor size on breast cancer death is also shown in the Kaplan-Meier plots of Figure 2a. The high number of patients with the heterogeneous grade 2 probably explains why grade is not as strong a prognostic marker as expected in this series of samples. In a multivariate Cox regression model obtained after elimination of all non-significant factors, the *TP53 *status, tumor size and lymph node status are the only significant remaining factors (Table 2, Multivariate analysis), and their effects are about the same as in the univariate analysis.

**Table 2:** Cox regression analysis for breast cancer death for all patients (*n *= 179 to 200)

	Univariate analysis			Multivariate analysis		
		
	RR	95 percent CI	*P*	RR	95 percent CI	*P*
Age, ≥55 years versus <55 years	0.86	0.51–1.44	0.57			
Tumor type (overall effect)			0.14			
Lobular (versus ductal)	0.56	0.30–1.06	0.074			
Other (versus ductal)	0.66	0.26–1.67	0.38			
Tumor size (overall effect)			0.001			0.002
pT2 (versus pT1)	2.22	1.17–4.20	0.014	1.94	0.99–3.82	0.054
pT3–pT4 (versus pT1)	4.17	1.93–9.01	0.0003	4.44	1.95–10.1	0.0004
Lymph node status (overall effect)			0.035			0.027
pN1 (versus pN0)	1.34	0.69–2.62	0.39	1.09	0.54–2.21	0.81
pN2–pN3 (versus pN0)	3.42	1.82–6.41	0.0001	2.73	1.41–5.30	0.003
Other^a ^(versus pN0)	1.69	0.77–3.67	0.19	1.28	0.51–3.20	0.60
Histological grade (overall effect)			0.47			
G2 (versus G1)	0.99	0.39–2.50	0.98			
G3 (versus G1)	1.43	0.52–3.90	0.49			
TP53 mutation (versus wild type)	4.51	2.69–7.56	<0.0001	5.24	3.03–9.07	<0.0001
ER positive (versus negative)	0.72	0.43–1.22	0.23			
PR positive (versus negative)	0.82	0.49–1.36	0.44			

^a^Lymph nodes not removed. 95 percent CI, 95 percent confidence interval for relative risk; ER, estrogen receptor; PR, progesterone receptor;*P*, *P *value for the hypothesis of no effect; RR, relative risk (hazard ratio).

### Survival analysis of patients with gene expression data

**Figure 3:**
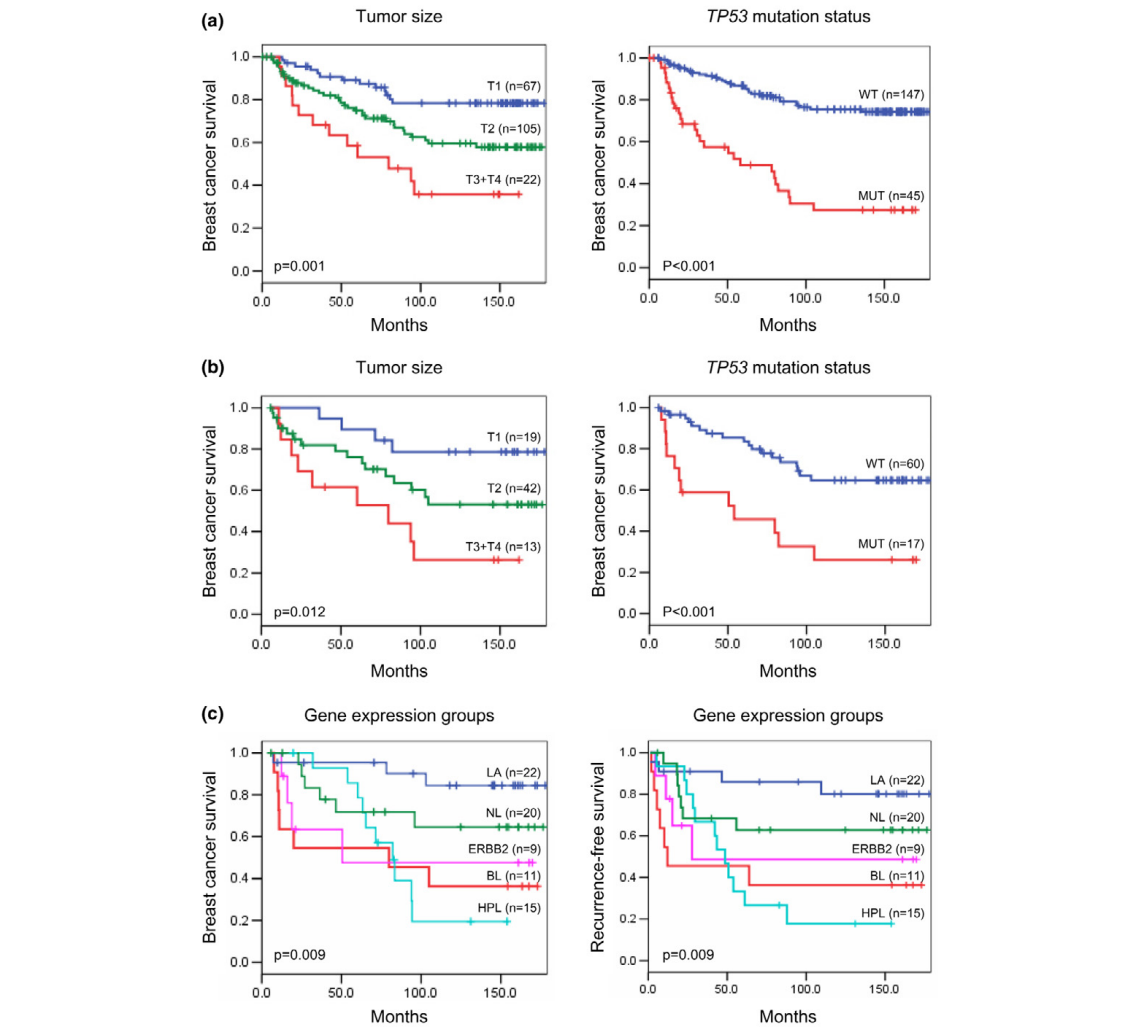
Kaplan-Meier survival curves. **(a) **Kaplan-Meier plots of breast cancer survival for all patients. The left panel shows tumor size (T1, T2, T3+T4) and the right panel *TP53 *mutation status (WT, wild type; MUT, mutation). **(b) **Kaplan-Meier plots of breast cancer survival for patients with gene expression data; the left panel shows tumor size and the right panel *TP53 *mutation status. **(c) **Kaplan-Meier plots of breast cancer survival and recurrence-free survival according to gene expression group (LA, luminal A; NL, normal-like; ERBB2; BL, basal-like; HPL, highly proliferating luminals). The *p *value from the log rank test (Mantel-Cox) is shown in each panel; 'n' is the number of samples in each group. Deaths due to causes not related to breast cancer were treated as censored observations.

By performing univariate Cox regressions on the samples included in the array experiment for all factors considered, *TP53 *mutation status, gene expression group, tumor size and lymph node status were all significant factors for survival (Table 3, Univariate analysis). The effect of tumor size is somewhat larger than for the analysis of all patients, while the effect of *TP53 *status is somewhat smaller. The gene expression groups have a large effect on survival. The breast cancer death rates for the highly proliferating luminal, the basal-like and the ERBB2^+ ^groups are about six times higher than for the luminal A group, while the breast cancer death rate for the normal-like group is almost three times that of the luminal A group. As the assumption of proportional hazards is violated for the highly proliferating luminals, the relative risk estimate for this group should be interpreted as an average over the time period considered. The effect of *TP53 *mutation status and tumor size on breast cancer death is also shown in the Kaplan-Meier plots in Figure 3b, while Figure 3c gives the Kaplan-Meier plot for the five gene expression groups. The non-proportionality of the hazard of highly proliferating luminals is clearly seen in the latter.

**Table 3:** Cox regression analysis for breast cancer death for patients with gene expression data (*n *= 69 to 77)

	Univariate analysis			Multivariate analysis		
		
	RR	95 percent CI	*P*	RR	95 percent CI	*P*
Age, ≥55 years versus <55 years	1.22	0.52–2.84	0.65			
Tumor type (overall effect)			0.32			
Lobular (versus ductal)	0.65	0.28–1.51	0.31			
Other (versus ductal)	0.34	0.05–2.53	0.29			
Tumor size (overall effect)			0.014			
pT2 (versus pT1)	2.63	0.88–7.86	0.085			
pT3–pT4 (versus pT1)	5.27	1.61–17.2	0.006			
Lymph node status (overall effect)			0.016			0.004
pN1 (versus pN0)	0.45	0.16–1.26	0.13	0.47	0.17–1.33	0.15
pN2–pN3 (versus pN0)	2.41	0.95–6.08	0.064	3.42	1.29–9.05	0.013
Other^a ^(versus pN0)	2.12	0.75–5.98	0.16	2.70	0.93–7.78	0.067
Histological grade (overall effect)			0.68			
G2 (versus G1)	2.20	0.30–16.3	0.44			
G3 (versus G1)	2.07	0.25–16.8	0.50			
TP53 mutation (versus wild type)	3.46	1.66–7.21	0.002	4.43	2.04–9.64	0.0004
ER positive (versus negative)	0.75	0.35–1.63	0.48			
PR positive (versus negative)	0.71	0.33–1.51	0.38			
Gene expression (overall effect)			0.006			
Highly proliferating luminal (versus luminal A)	6.59	1.79–24.3	0.005			
Normal like (versus luminal A)	2.82	0.71–11.3	0.14			
Basal like (versus luminal A)	6.93	1.79–26.8	0.005			
ERRB2^+ ^(versus luminal A)	5.82	1.30–26.2	0.022			

^a^Lymph nodes not removed. 95 percent CI, 95 percent confidence interval for relative risk; ER, estrogen receptor; PR, progesterone receptor; *P*, *P *value for the hypothesis of no effect; RR, relative risk (hazard ratio).

In a multivariate Cox regression model obtained after elimination of all non-significant factors, *TP53 *mutation status and lymph node status are the significant remaining factors (Table 3, Multivariate analysis). Gene expression groups and tumor size were the last factors to be eliminated. *TP53 *mutation status and gene expression group are closely related; 17 of the 20 patients with *TP53 *mutation are in the basal-like or ERBB2^+ ^group. This makes it difficult to separate the effect of *TP53 *from the effect of the gene expression groups. *TP53 *mutation and lymph node status are the strongest predictors of survival in the multivariate analysis, with effects of about the same size as in the univariate analyses.

### Three previously published gene lists as classifiers

Our set of samples was also classified according to three previously described gene lists [4,8,9]. By clustering the samples using the genes overlapping with our arrays, the samples were separated into two main branches in each dendrogram; predicted to be a good prognosis group and a poor prognosis group. Although the genes on our arrays did not overlap with all genes from the respective lists and the respective score procedure in each paper was not followed, it was interesting to see whether the gene lists had significance using this simple approach. In univariate Cox regression analysis two of the gene lists were significant in predicting breast cancer survival (15/21 genes: relative risk (RR) 3.70, 95%CI 1.64 to 8.34, *P *= 0.0007; 63/76 genes: RR 2.24, 95%CI 1.09–4.61, *P *= 0.028), while the third was close to being significant despite the limited number of overlapping genes (26/70 genes: RR 1.99, 95%CI 0.93 to 4.26, *P *= 0.067). None of the classifiers were significant when included in multivariate analysis together with the variables listed in Table 3, a result where the interpretation should be made according to the previous stated limitations of our approach.

### *TP53 *status in basal-like and ERBB2^+ ^carcinomas

**Figure 4:**
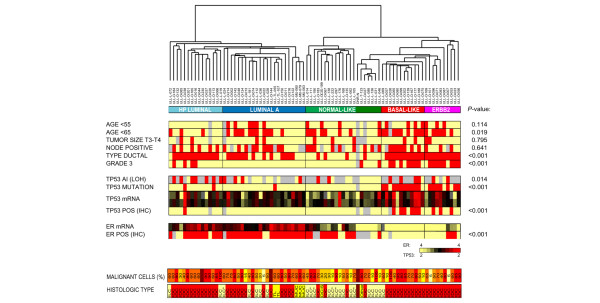
Clinical, histopathological and molecular characteristics of subgroups. Dendrogram from hierarchical clustering with distribution of clinical, histopathological and molecular markers between the five gene expression groups (highly proliferating luminals, luminal A, normal-like, basal-like, and ERBB2^+^). The color coding is as follows: red, description to the left; gray, unknown; yellow, all other. *P *values from cross-tabulation and Pearson *X*^2^-test are shown to the right of the panel. Relative expression of mRNA is shown for *TP53 *(Clone-ID: IMAGE:24415 and IMAGE:236338) and estrogen receptor (ER) (IMAGE:725321). The fraction of malignant cells in tumor tissue and histological type are shown in the lower panel: DCIS, ductal carcinoma *in situ *with microinvasion IDC, invasive ductal carcinoma; ILC, invasive lobular carcinoma; MPC, metaplastic carcinoma; MUC, mucinous carcinoma; TLC, tubulolobular carcinoma. ILC* is the 'typical lobular' type previously described [36]. IHC, immunohistochemistry; LOH, loss of heterozygosity.

The most striking characteristic of the basal-like and ERBB2^+ ^subclusters (Figure 4) was that most cases carried a *TP53 *mutation in their tumor. In the basal-like dendrogram branch 83% (10/12) of the carcinomas harbored a *TP53 *mutation, and in the ERBB2^+ ^subcluster the fraction was 70% (7/10). In the two basal-like tumors with wild-type *TP53 *(Figure 4) the mutations may have been missed, or other components of the pathway may have been affected. The basal-like subcluster showed a higher correlation between the samples in the dendrogram compared to other subclusters, as well as higher correlation to the 'basal-like centroid' (Figure 2), which may reflect the strong impact of a *TP53 *mutation on the global transcription program.

Most of the *TP53 *mutations detected among the 80 samples analyzed on the microarray (17/20, 85%) and among the total series of samples (39/48, 81%) were missense mutations, which is the type of mutation most frequently found in *TP53 *[34]. Only three samples outside the basal-like and ERBB2^+ ^clusters were affected with *TP53 *mutations; two missense mutations outside the DNA binding domain (codon 113, codon 138) affecting the highly proliferating luminals and one frequent missense mutation in the DNA major groove interacting domain (codon 273) affecting the luminal A group. Figure 4 further shows that IHC detected only 50% (10/20) of the mutations detected by TTGE. Almost half of the samples analyzed had AI (LOH) of *TP53 *in their tumor tissue (array: 19/41, 46%; total: 47/98, 48%), and LOH was strongly associated with the presence of a *TP53 *mutation (*p *< 0.001). Among the samples analyzed using microarray analysis, the samples with the highest frequency of AI seemed to cluster in the outermost three subgroups with the poorest outcome (basal-like, ERBB2^+ ^and highly proliferating luminal groups). Interestingly, the highly proliferating luminals showed a high frequency of AI (7/8, 88%) despite a low frequency of mutations in the *TP53 *gene.

Although the relative expression level of *TP53 *mRNA measured using microarrays showed variation between individual samples, the basal-like and ERBB2^+ ^groups, which had the most *TP53 *mutants, clearly showed the highest mRNA expression, while the luminal group had intermediate expression and the normal-like group had a lower expression (the data were centered to median expression across the dataset; Figure 4). Two of the three mutated samples falling outside the basal-like and ERBB2^+ ^groups had mutations that gave a lower relative expression of *TP53 *mRNA.

### Genes related to mutant *TP53*

**Figure 5:**
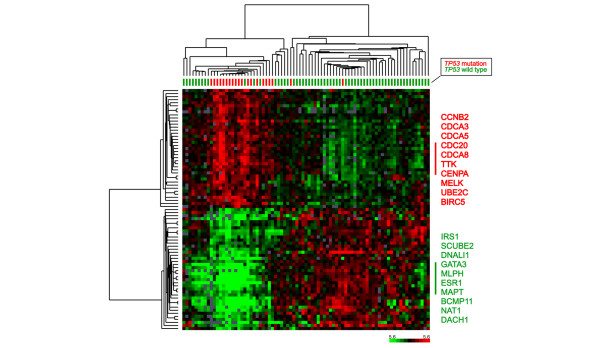
Gene expression pattern associated with *TP53 *mutations status. Hierarchical clustering of 80 samples using 80 genes selected by significance analysis of microarray analysis to be associated with *TP53 *mutation status. Tumor samples with *TP53 *mutation are labeled red and wild-type samples are labeled green (upper dendrogram). Ten genes with the highest correlation in each of the two main branches of the gene cluster (left dendrogram) are listed on the right.

SAM analysis was performed to find the gene expression pattern specific for tumors containing a *TP53 *mutation. With an estimated median number of false negatives being zero (delta slider 1.56 and fold change 2.0), 377 significant clones were selected when analyzing a set of 27,393 cDNA clones (Additional file 3). The highly specific gene expression pattern associated with *TP53 *mutation status is illustrated in Figure 5, where hierarchical clustering of the 80 samples and 80 selected genes (the 40 most significantly upregulated and 40 most downregulated genes of the 377 genes) are shown. Many genes that showed higher relative expression in carcinomas with a *TP53 *mutation were involved in the cell cycle and cell proliferation (for example, *CCNB2*, *CDCA5*, *CENPA*, and *UBE2C*), while the genes with lower relative expression showed more diverse functions and were highly associated with ER status (for example, *IRS1, ESR1, DNAL1 *and *NAT1*). Some of the genes (for example, *MYBL2, CDCA8, DNALI1 *and *DACH1*) were also identified recently by Miller and colleagues [35] as part of a 32-gene expression signature that distinguishes *TP53 *mutant and wild-type tumors. Further investigation of the gene expression pattern of different *TP53 *mutations is needed to understand more about the different effects they have in breast cancer.

### Relationship between clinicopathological markers and subgroups

The distribution of clinicopathological markers between the gene expression subgroups is shown in Figure 4. The subgroups that showed the poorest survival had the largest difference in median age, members of the basal-like and ERBB2^+ ^groups being the youngest (median age 56 years (28 to 75) and 60 years (47 to 87), respectively) and those of the highly proliferating luminal group the oldest (median age 75 years (59 to 82)). Dividing the patients into two equally large groups (<65 years and ≥65 years) gave a significant skewed distribution between the five gene expression groups (*p *= 0.019), but there was no significant difference when dividing them into pre- and post-menopausal women (<55 years and ≥55 years) (Figure 4). Tumor size did not show a statistically significant skewed distribution between the gene expression groups, suggesting that the gene expression patterns provide new and different information compared to this established marker. Neither was node status associated with any particular gene expression group.

The histological type and grade, as well as ER and *TP53 *mutation status all showed a highly significant skewed distribution between the gene expression groups (*p *< 0.001; Figure 4). The ERBB2^+ ^and basal-like groups showed the highest fraction of grade 3 tumors (80% and 58%, respectively). Although carcinomas of the luminal A and highly proliferating luminal subgroups were mainly grade 2, they differed by the fact that the luminal A group included 22% of cases with grade 1 and no grade 3 cases, while the highly proliferating luminal group included 27% of cases with grade 3 and no grade 1 cases. Among the luminal samples, 89% were ER positive, while none of the basal-like and only 30% of the ERBB2^+^-group were ER positive. The relative mRNA expression of ER (centered over the dataset) was compared to protein expression (IHC) and showed a strong correlation (Figure 4).

### Tumor cell content and histological types

Tissue samples with a low fraction of malignant epithelial cells were included in this study to increase our understanding of how this may affect a tumor's characteristic gene expression pattern. Samples with different percentages of malignant epithelial cells were distributed among all subclasses, although an over-representation (not statistically significant) of tumors with low tumor content were seen in the normal-like subgroups (Figure 4). The invasive lobular carcinomas tended to have lower tumor content than invasive ductal carcinomas. Hierarchical clustering of samples with ≥40% tumor cells results in the same subgroups as when all samples were included. These results suggest that the gene expression profile of a grossly dissected tumor sample in many cases is not heavily influenced by a relatively low quantity of malignant cells, and that tumor stromal cells also may affect the gene expression profile.

Invasive ductal carcinomas were distributed among all subclusters, but entirely dominated the highly proliferating luminal, basal-like and ERBB2^+ ^groups (Figure 4). Invasive lobular carcinomas were divided into two groups, those clustering with the normal-like subgroup, previously referred to as 'typical-lobular' [36], and the 'ductal-like lobular' clustering with the two luminal groups (14/22 and 7/22, respectively). The only lobular samples clustering on the edge of the basal-like group were alveolar lobular with pleomorphic areas. Two tubulolobular carcinomas clustered together in the highly proliferating luminal subgroup, and three mucinous carcinomas clustered together in the luminal A subgroup, showing that the phenotypes distinguished by pathologists are also distinct on a molecular level. A fourth sample, an invasive ductal carcinoma, was part of the 'mucinous cluster,' showing a gene expression pattern similar to these tumors despite the fact that no typical mucinous elements in paraffin-embedded or frozen tissue sections from this patient were seen. The mucinous samples were all ER positive by IHC, *TP53 *wild type, ERBB2 negative and E-cadherin positive. A ductal carcinoma *in situ *with microinvasion and a metaplastic carcinoma clustered in the normal-like subgroup. The invasive ductal carcinoma sample from a man (ULL-020) clustered with the highly proliferating luminal group.

## Discussion

In the patient series analyzed here both uni- and multivariate analysis show that *TP53 *mutation status was a very pronounced prognostic factor. Although some studies have reported similar findings, others have found a weaker prognostic power for *TP53 *mutation status [37], which may be due to the mutation screening approach used (as well as population differences). The most frequently used method for mutation screening of the *TP53 *gene has been IHC, which detects only mutations that induce protein accumulation, missing frameshift, nonsense and splice mutations. In this study, several of the missense mutations with high levels of mRNA expression were also missed by IHC (Figure 4), showing the insufficiency of this technique for mutation screening. The TTGE/sequencing analysis detected 15% of the *TP53 *mutations outside exons 5 to 8, supporting the importance of analyzing the whole gene and not only exons 5 to 8 as many previous studies have done.

A key issue is whether *TP53 *mutation status is a prognostic marker or instead a marker of therapy response only (predictive marker). The results in Table 2 (Multivariate analysis) show the total effects of tumor size and *TP53 *status on survival, effects that may be direct and/or indirect via adjuvant treatment. When including adjuvant therapy in the multivariate analysis, RR values similar to those in Table 2 were found (*TP53*, RR 5.1), indicating that the total effects are mainly a result of the direct effects, not indirect effects via treatment. Analysis of patients receiving surgery only (no adjuvant treatment) also gave similar result (*TP53*, RR 4.3). Although several studies have suggested that *TP53 *mutation status is a predictive factor [38,39], randomized large-scale studies are needed to make certain of this. *TP53 *mutation status may be both a predictive marker of some treatment regimes as well as a strong prognostic factor.

The strong correlation of *TP53 *mutations with the basal-like subtype is a biologically important finding, and whether it is the nature of ER-negative basal-like tumors that allows mutational events in the *TP53 *gene or that the basal-like gene expression profile is a consequence only of a *TP53 *mutation is unresolved and should stimulate further investigation on the origin of breast tumor cells. A related question to address in larger studies is whether the specific gene expression pattern we found associated with *TP53 *mutation status was a result of cellular events directly initiated by mutant *TP53 *or rather a result of the dominant cell type (basal-like progenitor or cancer stem cell) in these tumors. Similar questions apply to the ERBB2^+ ^subtype, which also shows a strong correlation with *TP53 *mutations; in addition, the sequence and impact of the ERBB2 amplification versus the *TP53 *mutational event needs investigation. Sørlie and colleagues [2] reported in their patient cohort of locally advanced breast cancer a high frequency of *TP53 *mutations also within the luminal B samples (highly proliferating luminal cases). A relatively low frequency of *TP53 *mutations was found within the highly proliferating luminals (2/15) in our set of patients with earlier stage tumors. We propose that *TP53 *mutations may be an early and causal event in basal-like tumors whereas in luminal B (highly proliferating luminals) tumors it may be a consequence of genomic instability. The strong association found between AI and point mutations in the *TP53 *gene in the basal-like and ERBB2^+ ^samples support the concept of *TP53 *acting as a tumor suppressor gene in these tumors [40], while the high frequency of AI despite a low frequency of *TP53 *mutations in the highly proliferating luminal group suggests a different mechanism for *TP53 *in these tumors.

There is a massive interest in defining gene expression profiles of breast tumors to understand the development and progression of the disease and to create a novel clinically useful diagnostic tool. Many reports are very promising, although the clinical and genetic heterogeneity of the disease does not make it straightforward to predict recurrence and outcome in individuals based on a snapshot of the biological processes in the individual tumor. Our study aimed to investigate the potential of gene expression profiling as a prognostic marker in patients with long term follow-up, and not to create yet another gene list associated with patient outcome. The extreme amount of variables (genes) and the relatively low number of cases and events increases the probability of accidental but apparently significant findings [41] in microarray analysis. In this study we have chosen an unsupervised approach for the classification of samples. The results certainly support the huge potential of information found in expression patterns, and the classification is shown to be a statistically highly significant predictor of survival.

The Kaplan-Meier plot (Figure 3c) illustrates a significant difference in survival between the different expression groups, as seen in previous studies [3]. Notice that the two groups with very poor prognosis had a diverse progression of disease. Breast cancer cases in both the basal-like and ERBB2^+ ^groups had a very high mortality rate during the first two years, while the highly proliferating luminal cases developed the disease more slowly, showing highest mortality after five to eight years. We were not able to pinpoint any specific heterogeneity (clinical, histopathological or molecular markers) of the patients within the highly proliferating luminal cluster, the group showing non-proportional hazard, and suggest the curve reflects biological characteristics. Many patients with highly proliferating luminal cancer received Tamoxifen treatment for two years, and the poor outcome in this group compared to luminal A patients could be explained by the lack of a Tamoxifen effect. Alternatively, this anti-estrogen treatment may temporarily prolong patient survival in this group for the first years they receive the drug. The different progression observed in basal-like versus highly proliferating luminal patients may be consistent with the bimodal mortality rate reported by Demicheli and colleagues [42].

Different approaches have been used in an attempt to define clinically relevant groups based on gene expression patterns, but a consensus on how to do this has not yet been reached. In our study a classification similar to the one identified by Sørlie and colleagues [2] was obtained, supporting the existence of such subgroups in a broader spectrum of breast tumor stages. A few samples were, for various reasons, difficult to categorize. The lack of proliferation genes in the intrinsic gene list causes a less clear correlation with the luminal B centroid, but when proliferation genes from the total cluster (Figure 1h) were included the characteristics of the highly proliferating luminals (luminal B-like) compared to the luminal A group were clearly shown. Although the majority of luminal samples were most highly correlated with the luminal A-centroid, the group we named highly proliferating luminals is clearly different from the luminal A group in the scatter chart, having the second highest correlation with the luminal B-centroid (Figure 2). We suggest that earlier stages of luminal B (here named highly proliferating luminals) may have less pronounced expression profiles than the advanced tumors where the centroids were defined (our data set versus Sørlie and colleagues [2]). The small cluster between the normal-like and the basal-like group shows highest correlation with the ERBB2-centroid, although this group demonstrates extremely low expression of both the ERBB2 gene (Figure 1g) and basal-like genes (Figure 1f). The samples seem more normal-like based on the fact that a normal breast tissue sample clustered within this group, as well as showing expression of genes previously identified to characterize normal-like samples. This small cluster illustrates the difficulties in assigning individual samples to subgroups based on correlation with centroids alone. The correlation of each sample with each of the centroids showed a continuous pattern over the sample set, and visualizes how each sample carries elements from different profiles (Figure 2). In Figure 1g, the ERBB2^+ ^group on the far right side shows high expression of an ERBB2-related gene cluster and is, therefore, included in this group, despite the fact that its members also show correlation with the luminal B centroid. It is a matter of choice which group to assign these samples to. The ERBB2^+ ^group is defined by a molecular event (overexpression of ERBB2), whereas the luminal B group is recognized by highly proliferating ER-positive tumors. Three samples do not show increased ERBB2 expression, but they are included in the ERBB2^+ ^group based on their clustering. Although these samples express a low level of ERBB2 on the RNA level, it has been observed that the protein level (fluorescence *in situ *hybridization analysis) does not always correspond and thus may be high.

## Conclusion

The combination of gene expression groups and clinical/histopathological parameters in this study has added more details and levels of understanding to our current picture of breast carcinomas. The long follow-up of patients revealed that the highly proliferating luminal group had an even worse prognosis than the basal-like and the ERBB2^+ ^groups. The relatively good outcome for the first five years for the highly proliferating luminal group may be explained by the natural history of these tumors or by use of Tamoxifen. The strong association found between the basal-like group and *TP53 *mutations suggests that such mutations may be causal in these tumors, while *TP53 *mutations may be a later event in the highly proliferating luminal carcinomas. The high frequency of *TP53 *AI in the highly proliferating luminal group supports a mechanism other than *TP53 *mutations causing genomic instability in these tumors, and should be further explored. The characteristic gene expression pattern found in tumors carrying a *TP53 *mutation also needs further investigation in larger sets of samples with various mutations included.

Both *TP53 *mutation status and gene expression subgroups demonstrated strong prognostic impact, and may add valuable new information that complements the established prognostic markers. *TP53 *may help distinguish high risk tumors in need of treatment from among small, node negative tumors, which do not currently receive adjuvant treatment (that is, they are undertreated); on the other hand, it may help avoid treatment of individuals in patient groups that today may be overtreated. The choice of treatment may, for example, be influenced by avoiding drugs dependent on *TP53*-mediated apoptosis or, in the future, by using drugs that target and reactivate *TP53*. Although gene expression-based subgroups showed massive prognostic strength, a more robust classification method is needed for future application in clinical practice. Development of a new integrated prognostic model that includes *TP53 *and gene expression groups could be useful in the choosing of treatment.

## Abbreviations

AI = allelic imbalance; CMF = cyclophosphamide, methotrexate, 5-fluorouracil; ER = estrogen receptor; IHC = immunohistochemistry; LOH = loss of heterozygosity; PR = progesterone receptor; RR = relative risk; SAM = significance analysis of microarray; TTGE = temporal temperature gradient gel electrophoresis.

## Competing interests

The authors declare that they have no competing interests.

## Authors' contributions

AL collected updated clinical data, participated in revising the histology, carried out the gene expression analysis, organized the *TP53 *mutation analysis, participated in the design of the study, performed some statistical analysis and drafted the manuscript. HZ carried out parts of the gene expression analysis. ØB participated in the design of the study and carried out the statistical survival analysis. JMN carried out the histology analysis. IRKB and TI collected clinical and molecular data. RK collected the patient material. ALBD conceived of the study, participated in its design and coordination and helped draft the manuscript. SSJ participated in the design of the study, coordinated the gene expression analysis and helped draft the manuscript. All authors read and approved the final manuscript.

## Supplementary Material

Additional file 1 A table listing clinical information (array). Click here for file

Additional file 3 A table listing TP53 associated genes. Click here for file

Additional file 2 A table listing TP53 mutations. Click here for file

Additional file 4 A figure showing the hierarchical clustering using the total set of genes. Click here for file
