# Supplementary material for: TP53 mutation status and gene expression profiles are powerful prognostic markers of breast cancer
Source: Breast Cancer Res. 2007 May 15;9(3):R30. doi: 10.1186/bcr1675 (PMC1929092; doi:10.1186/bcr1675)
Supplement: Additional file 1 — A table listing clinical information (array). [file bcr1675-S1.pdf]

Table S1. Clinical information (array)

**Patient ID**	**Age onset** (year)	**Recurrence** [0=no, 1=local, 2=distant (+/-local)]	**Recurrence code** (distant metastasis or breast ca death) (0=no, 1=yes, 9=unknown)	**Status** (0=alive, 1=dead of breast ca., 2=dead other cause, 3=emigrated, 9=unknown)	**Recurrence free survival** (months; first distant metastasis or breast cancer death)	**Breast cancer survival** (months)	**Histological type**	**Tumor size** (1-4=T1-T4, 9=unknown)	**Node category** (0=neg, 1=pos, 9=unknown)	**Metastasis** (0=no, 1=yes)	**Grade** (1-3)	**TP53 mut** (0=wt, 1=mut)	**ER** (0=neg, 1=pos, 9=unknown)	**PR** (0=neg, 1=pos, 9=unknown)	**Adjuvant chemotherapy** (0=no, 1=yes, 9=unknown)	**Adjuvant radiation therapy** (0=no, 1=yes, 9=unknown)	**Adjuvant hormonal therapy** (0=no, 1=yes, 9=unknown)	**Gene expression subgroups** (1=luminal A, 2=highly proliferating luminals, 3=normal-like, 4=basal-like, 5=ERBB2
ULL - 002	87.7	0	0	2	9.8	9.8	Ductal	2	9	0	2	0	1	1	0	0	1	1
ULL - 007	86.9	2	1	1	27.7	50.5	Ductal	1	9	0	2	1	1	1	0	0	0	5
ULL - 011	78.8	0	0	2	145.5	145.5	Ductal	2	0	0	2	0	1	0	0	0	0	1
ULL - 013	76.1	0	0	2	70.4	70.4	Ductal	2	1	0	1	0	1	1	0	0	1	1
ULL - 014	46.8	2	1	1	19.4	46.5	Lobular	2	1	0	2	0	1	1	0	0	1	3
ULL - 016	67.3	0	0	2	131.1	131.1	Ductal	1	0	0	3	0	1	1	0	0	0	2
ULL - 019	81.8	2	1	2	48.7	72.8	Lobular	2	0	1	2	0	1	1	0	0	0	2
ULL - 020	75.8	2	1	1	87.8	93.9	Ductal	3	1	0	2	0	9	9	0	1	1	2
ULL - 022	28.3	1	0	3	146.1	146.1	Ductal	3	1	0	2	0	0	0	0	1	1	1
ULL - 023	73.7	0	9	9	164.6	164.6	Ductal	2	0	0	3	1	1	1	0	0	0	5
ULL - 024	74.2	1	0	2	144.4	144.4	Lobular	9	1	0	2	0	9	9	0	1	0	1
ULL - 026	40.3	0	0	0	173.0	173.0	Ductal	1	1	0	3	0	9	9	1	1	0	4
ULL - 027	71.3	2	1	1	18.5	27.0	Ductal	9	1	0	2	0	9	9	0	0	1	3
ULL - 028	62.6	0	0	0	177.9	177.9	Lobular	1	1	0	2	0	0	1	0	0	0	1
ULL - 031	84.3	1	0	2	70.2	70.2	Lobular	2	9	0	2	0	0	1	0	0	0	3
ULL - 036	70.6	1	0	2	117.9	117.9	Lobular	1	0	0	2	0	1	1	0	9	0	1
ULL - 037	78.2	2	1	1	50.8	83.4	Ductal	2	0	0	2	0	1	1	0	0	0	2
ULL - 038	74.8	2	1	1	61.1	82.2	Ductal	1	0	0	3	1	1	1	0	1	0	2
ULL - 044	81.9	2	1	1	22.9	71.5	Ductal	1	1	0	2	0	1	1	0	0	0	2
ULL - 046	41.2	0	0	0	176.5	176.5	Lobular	2	1	0	3	0	1	1	1	0	1	3
ULL - 048	61.0	2	1	0	109.5	172.8	Ductal	2	1	0	2	0	1	1	0	1	1	1
ULL - 053	62.4	0	0	2	13.6	13.6	Ductal	2	1	0	3	0	1	1	0	1	1	5
ULL - 055	80.9	0	9	9	129.8	129.8	Tubulolobular	1	9	0	1	1	1	1	0	0	0	1
ULL - 056	76.6	2	1	1	11.2	16.1	Ductal	2	0	0	3	1	0	1	0	0	0	5
ULL - 057	47.5	2	1	1	7.3	7.7	Ductal	2	9	0	2	1	0	1	1	1	0	4
ULL - 060	74.7	0	0	2	145.5	145.5	Mucinous	2	0	0	2	0	1	1	0	0	0	1
ULL - 062	62.1	0	0	0	160.9	160.9	Lobular	2	0	0	2	0	1	1	0	1	0	3
ULL - 063	80.8	2	1	1	6.6	7.1	Mucinous	2	9	0	1	0	1	0	0	0	0	1
ULL - 065	59.5	0	0	0	167.6	167.6	Ductal	2	1	0	3	1	0	1	1	1	1	4
ULL - 066	78.4	2	1	2	5.2	19.7	Ductal	2	1	0	2	0	1	1	0	0	0	2
ULL - 067	62.2	2	1	1	18.2	25.0	Ductal	2	0	0	2	0	1	0	0	0	0	3
ULL - 069	56.1	2	1	1	5.5	20.1	Lobular	2	0	0	2	1	0	1	0	0	0	4
ULL - 071	48.4	0	0	0	167.7	167.7	Ductal	2	0	0	3	1	0	1	0	0	0	5
ULL - 072	58.7	2	1	1	42.1	65.3	Lobular	2	9	0	2	0	1	0	0	1	1	2
ULL - 074	43.2	0	0	0	163.5	163.5	Ductal	2	1	0	2	0	1	1	1	1	1	1
ULL - 075	28.2	0	0	0	163.4	163.4	Ductal	2	0	0	2	0	0	0	0	0	0	4
ULL - 079	86.1	0	0	2	95.0	95.0	Mucinous	2	9	0	2	0	1	0	0	0	0	1
ULL - 080	64.5	2	1	1	12.1	104.9	Ductal	2	1	0	2	1	0	0	0	0	0	4
ULL - 083	58.3	0	0	3	21.2	21.2	Ductal	2	1	0	3	1	0	1	1	1	0	5
ULL - 085	59.4	2	1	1	29.5	32.0	Ductal	4	1	0	2	0	0	1	9	1	1	2
ULL - 087	47.4	0	0	0	161.0	161.0	Ductal	1	0	0	3	0	0	1	0	0	0	5
ULL - 088	70.9	0	0	0	161.1	161.1	Lobular	2	0	0	2	0	0	0	0	9	0	3
ULL - 096	57.0	0	0	0	169.8	169.8	Ductal	2	0	0	3	1	0	0	1	9	0	5
ULL - 097	82.4	0	0	2	5.8	5.8	Lobular	2	9	0	2	0	1	1	0	0	0	3
ULL - 099	55.1	0	9	9	110.8	110.8	Ductal	2	1	0	3	1	0	1	0	1	1	4
ULL - 101	81.0	2	1	1	15.3	18.9	Ductal	4	0	0	3	1	0	1	0	0	0	5
ULL - 105	73.7	2	1	1	55.8	95.9	Lobular	3	1	0	2	0	1	1	0	1	1	3
ULL - 107	71.9	0	0	2	122.1	122.1	Tubulolobular	1	1	0	1	0	1	1	0	0	0	1
ULL - 111	55.3	0	0	0	171.2	171.2	Lobular	1	1	0	2	0	1	1	0	1	1	3
ULL - 112	46.6	0	0	0	161.9	161.9	Lobular	3	1	0	1	0	1	1	1	0	1	1
ULL - 113	65.6	2	1	1	28.3	53.9	Ductal	2	0	0	2	1	1	1	0	0	0	2
ULL - 122	72.5	0	0	0	160.6	160.6	Ductal	1	0	0	2	0	1	1	0	0	0	1
ULL - 123	75.2	1	0	2	40.0	40.0	Other	4	0	0	3	0	9	0	9	0	9	3
ULL - 132	69.5	0	0	2	83.0	83.0	Ductal	9	0	0	2	0	0	0	0	0	0	2
ULL - 134	60.3	0	0	0	171.5	171.5	Ductal	2	0	0	2	0	1	1	0	0	0	1
ULL - 135	73.3	0	0	0	167.3	167.3	Ductal	1	0	0	2	0	1	1	0	1	0	3

Table S1. Clinical information (array)

**Patient ID**	**Age onset** (year)	**Recurrence** [0=no, 1=local, 2=distant (+/-local)]	**Recurrence code** (distant metastasis or breast ca death) (0=no, 1=yes, 9=unknown)	**Status** (0=alive, 1=dead of breast ca., 2=dead other cause, 3=emigrated, 9=unknown)	**Recurrence free survival** (months; first distant metastasis or breast cancer death)	**Breast cancer survival** (months)	**Histological type**	**Tumor size** (1-4=T1-T4, 9=unknown)	**Node category** (0=neg, 1=pos, 9=unknown)	**Metastasis** (0=no, 1=yes)	**Grade** (1-3)	**TP53 mut** (0=wt, 1=mut)	**ER** (0=neg, 1=pos, 9=unknown)	**PR** (0=neg, 1=pos, 9=unknown)	**Adjuvant chemotherapy** (0=no, 1=yes, 9=unknown)	**Adjuvant radiation therapy** (0=no, 1=yes, 9=unknown)	**Adjuvant hormonal therapy** (0=no, 1=yes, 9=unknown)	**Gene expression subgroups** (1=luminal A, 2=highly proliferating luminals, 3=normal-like, 4=basal-like, 5=ERBB2
ULL - 136	67.4	0	0	2	124.8	124.8	Lobular	2	1	0	2	0	0	1	1	1	0	3
ULL - 138	74.3	2	1	1	21.8	36.4	Lobular	1	0	0	2	0	1	0	0	0	0	3
ULL - 139	48.1	2	1	1	1.9	10.7	Ductal	2	0	0	3	1	0	0	0	0	0	4
ULL - 143	48.0	2	1	1	46.6	103.0	Ductal	2	0	0	2	0	1	1	0	0	0	1
ULL - 144	61.9	0	0	0	159.0	159.0	Ductal	1	1	0	2	0	1	1	0	0	1	1
ULL - 150	63.7	0	0	0	157.8	157.8	Ductal	1	1	0	2	0	1	1	0	0	1	1
ULL - 165	76.0	2	1	1	53.9	60.2	Ductal	4	1	0	3	0	1	1	0	1	1	2
ULL - 167	74.8	1	1	1	10.1	10.1	Ductal	2	1	0	3	1	0	0	0	1	0	4
ULL - 168	78.4	2	1	2	9.6	12.9	Lobular	2	9	0	2	0	0	0	0	0	1	3
ULL - 169	51.1	2	1	1	4.4	12.3	Ductal	3	1	0	2	0	0	0	1	1	0	5
ULL - 176	46.4	0	0	0	154.8	154.8	Lobular	2	1	0	1	0	0	0	1	0	0	3
ULL - 177	46.8	2	1	1	63.9	79.8	Ductal	3	0	0	3	1	0	0	0	0	0	4
ULL - 181	50.2	2	1	1	1.2	78.2	Lobular	2	9	1	2	0	1	1	0	0	0	1
ULL - 183	73.1	0	0	2	77.3	77.3	Ductal	1	0	0	2	0	9	0	0	0	0	3
ULL - 184	70.9	0	0	0	153.9	153.9	Ductal	1	1	0	3	0	9	0	0	1	1	2
ULL - 188	58.1	0	0	0	154.3	154.3	Ductal	2	1	0	3	1	0	0	0	0	0	4
ULL - 190	62.5	2	1	1	20.6	23.0	Lobular	3	1	0	2	0	9	0	0	0	1	3
ULL - 199	54.2	0	0	0	153.5	153.5	DCIS	1	9	0	2	0	1	0	0	0	0	3
ULL - 201	79.3	2	1	1	24.3	63.3	Ductal	2	0	0	2	0	1	1	0	0	1	2
ULL - 202	72.8	2	1	1	3.7	10.9	Ductal	4	1	0	2	1	0	0	0	1	0	4
ULL - 214	43.8	0	0	0	150.7	150.7	Ductal	1	0	0	2	0	1	1	0	0	0	1
ULL - 216	67.2	0	0	2	26.4	26.4	Ductal	2	0	0	2	0	1	1	0	9	0	1
ULL - 222	75.2	0	0	0	148.9	148.9	Lobular	3	1	0	2	0	1	1	0	1	1	3
ULL - 230	64.0	2	1	1	43.4	94.3	Ductal	2	0	0	2	0	1	1	0	0	0	2
