# Supplementary material for: TP53 mutation status and gene expression profiles are powerful prognostic markers of breast cancer
Source: Breast Cancer Res. 2007 May 15;9(3):R30. doi: 10.1186/bcr1675 (PMC1929092; doi:10.1186/bcr1675)
Supplement: Additional file 3 — A table listing TP53 associated genes. [file bcr1675-S3.pdf]

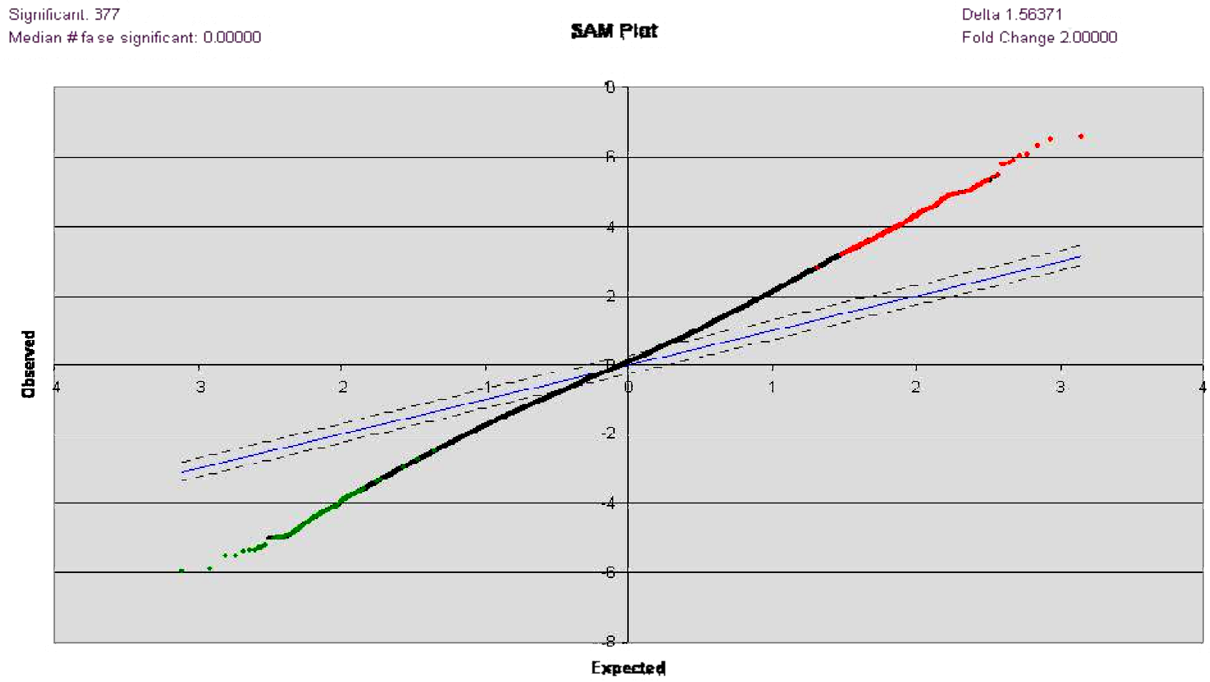

Table S3: Genes assiciated with *TP53* mutation status

242 Positive Significant Genes

Gene Name	Gene ID
118618 || ||	IMAGE:430186
112817 || CDCA5 || cell division cycle associated 5	IMAGE:296155
120329 || WDR5 || WD repeat domain 5	IMAGE:731023
310383 || CENPA || centromere protein A, 17kDa	IMAGE:2017415
110227 || BIRC5 || baculoviral IAP repeat-containing 5 (survivin)	IMAGE:796694
117963 || IBA2 || ionized calcium binding adapter molecule 2	IMAGE:178922
310062 || D21S2056E || DNA segment on chromosome 21 (unique) 2056 expressed sequence	IMAGE:2018941
314040 || MGC2408 || hypothetical protein MGC2408	IMAGE:1868534
102750 || GTPBP4 || GTP binding protein 4	IMAGE:230235
222658 || HSP70-4 || likely ortholog of mouse heat shock protein, 70 kDa 4	IMAGE:813999
99561 || CDCA8 || cell division cycle associated 8	IMAGE:292936
308729 || TTK || TTK protein kinase	IMAGE:2062329
107203 || LOC157378 || hypothetical protein BC017881	IMAGE:811020
98784 || AMD1 || adenosylmethionine decarboxylase 1	IMAGE:149013
116679 || SYNCRIP || synaptotagmin binding, cytoplasmic RNA interacting protein	IMAGE:753322
330790 || CCNB2 || cyclin B2	IMAGE:2457449
331070 || SLC7A5 || solute carrier family 7 (cationic amino acid transporter, y+ system), member 5	IMAGE:2541203
111783 || CDCA7 || cell division cycle associated 7	IMAGE:753198
108294 || LMO4 || LIM domain only 4	IMAGE:162533
223334 || CCNB2 || cyclin B2	IMAGE:856289
178439 || MELK || maternal embryonic leucine zipper kinase	IMAGE:327289
117492 || IFRD1 || interferon-related developmental regulator 1	IMAGE:121948
115756 || TK1 || thymidine kinase 1, soluble	IMAGE:379920
308749 || KIFC1 || kinesin family member C1	IMAGE:129961

225262 || || Homo sapiens mRNA similar to RIKEN cDNA 2700049P18 gene (cDNA clone MGC:57827 IMAGE:60	IMAGE:1035796
223867 || ARL6IP2 || ADP-ribosylation-like factor 6 interacting protein 2	IMAGE:743987
119447 || TRIP8 || thyroid hormone receptor interactor 8	IMAGE:773208
104988 || PFKP || phosphofructokinase, platelet	IMAGE:950682
105833 || KPNA2 || karyopherin alpha 2 (RAG cohort 1, importin alpha 1)	IMAGE:882510
330756 || LAMP3 || lysosomal-associated membrane protein 3	IMAGE:2239304
226162 || MELK || maternal embryonic leucine zipper kinase	IMAGE:1517595
312244 || ||	IMAGE:1702742
319961 || LRP11 || low density lipoprotein receptor-related protein 11	IMAGE:1867933
313311 || MCM7 || MCM7 minichromosome maintenance deficient 7 (S. cerevisiae)	IMAGE:2325609
102739 || PRO2000 || PRO2000 protein	IMAGE:204483
108697 || C20orf129 || chromosome 20 open reading frame 129	IMAGE:200402
99392 || CDC20 || CDC20 cell division cycle 20 homolog (S. cerevisiae)	IMAGE:898062
222270 || CDCA3 || cell division cycle associated 3	IMAGE:743810
107438 || LOC91526 || hypothetical protein DKFZp434D2328	IMAGE:292920
119851 || UBE2C || ubiquitin-conjugating enzyme E2C	IMAGE:769921
108023 || FLJ14502 || TRAF4 associated factor 1	IMAGE:111812
113261 || FOXM1 || **forkhead box M1	IMAGE:564803
110859 || EIF2C2 || eukaryotic translation initiation factor 2C, 2	IMAGE:129840
228997 || NUSAP1 || nucleolar and spindle associated protein 1	IMAGE:2327739
106519 || NDRG1 || N-myc downstream regulated gene 1	IMAGE:842863
98779 || ATP6V1C2 || ATPase, H+ transporting, lysosomal 42kDa, V1 subunit C isoform 2	IMAGE:123627
223871 || UBE2C || ubiquitin-conjugating enzyme E2C	IMAGE:146882
101932 || NP || nucleoside phosphorylase	IMAGE:769890
116712 || C10orf3 || chromosome 10 open reading frame 3	IMAGE:504308
115397 || CENPF || centromere protein F, 350/400ka (mitosin)	IMAGE:435076
103893 || AMD1 || adenosylmethionine decarboxylase 1	IMAGE:825842
105550 || IMPA2 || inositol(myo)-1(or 4)-monophosphatase 2	IMAGE:32299
310652 || DKFZp762E1312 || hypothetical protein DKFZp762E1312	IMAGE:1540236
111977 || FLJ10156 || hypothetical protein FLJ10156	IMAGE:773147
112622 || LGN || LGN protein	IMAGE:415264
223443 || FLJ10407 || hypothetical protein FLJ10407	IMAGE:824126
225799 || HAPIP || huntingtin-associated protein interacting protein (duo)	IMAGE:971279
310388 || TPX2 || TPX2, microtubule-associated protein homolog (Xenopus laevis)	IMAGE:1540227
108149 || EZH2 || enhancer of zeste homolog 2 (Drosophila)	IMAGE:770992
108920 || ANAPC7 || anaphase-promoting complex subunit 7	IMAGE:788256
108090 || PRO2000 || PRO2000 protein	IMAGE:280375
224310 || PKP1 || plakophilin 1 (ectodermal dysplasia/skin fragility syndrome)	IMAGE:399577
109251 || RRM2 || ribonucleotide reductase M2 polypeptide	IMAGE:624627
111792 || PLK || polo-like kinase (Drosophila)	IMAGE:744047
222476 || || Homo sapiens, clone IMAGE:5736845, mRNA	IMAGE:449112
98808 || || Homo sapiens cDNA clone IMAGE:4448513, partial cds	IMAGE:418279
225985 || CLIC4 || chloride intracellular channel 4	IMAGE:448085
109732 || RAD51 || RAD51 homolog (RecA homolog, E. coli) (S. cerevisiae)	IMAGE:1476053
331258 || EVA1 || epithelial V-like antigen 1	IMAGE:453112
224459 || PSAT1 || phosphoserine aminotransferase 1	IMAGE:1636108
105293 || RTP801 || HIF-1 responsive RTP801	IMAGE:221707
320000 || BCL11A || B-cell CLL/lymphoma 11A (zinc finger protein)	IMAGE:1584563
115359 || SHAPY || **Ca2+-dependent endoplasmic reticulum nucleoside diphosphatase	IMAGE:257011
308722 || FLJ25416 || hypothetical protein FLJ25416	IMAGE:1618978
118420 || TRIM2 || tripartite motif-containing 2	IMAGE:838899

220935 || PRKX || protein kinase, X-linked	IMAGE:1638550
100967 || KCNN4 || potassium intermediate/small conductance calcium-activated channel, subfamily N, member 4	IMAGE:756708
224556 || UBQLN1 || ubiquilin 1	IMAGE:824719
104095 || MYBL2 || v-myb myeloblastosis viral oncogene homolog (avian)-like 2	IMAGE:815526
223936 || PLEKHG1 || pleckstrin homology domain containing, family G (with RhoGef domain) member 1	IMAGE:298021
223533 || FLJ20989 || hypothetical protein FLJ20989	IMAGE:430549
108164 || KIFC1 || kinesin family member C1	IMAGE:292933
221572 || HMGB3 || high-mobility group box 3	IMAGE:878640
99680 || LOC89958 || hypothetical protein LOC89958	IMAGE:511096
162324 || || Homo sapiens transcribed sequences	IMAGE:712292
109438 || SLC7A5 || solute carrier family 7 (cationic amino acid transporter, y+ system), member 5	IMAGE:755578
104234 || SEPHS1 || selenophosphate synthetase 1	IMAGE:840702
107752 || CDC42EP1 || CDC42 effector protein (Rho GTPase binding) 1	IMAGE:214982
310078 || MGC5528 || defective in sister chromatid cohesion homolog 1 (S. cerevisiae)	IMAGE:1394099
221272 || EVA1 || epithelial V-like antigen 1	IMAGE:233464
224655 || || Homo sapiens cDNA FLJ16029 fis, clone KIDNE2012945, weakly similar to PROCOLLAGEN C-PROT	IMAGE:259884
109369 || CDC25B || cell division cycle 25B	IMAGE:48398
100181 || CTL2 || CTL2 gene	IMAGE:137581
102735 || B3GNT5 || UDP-GlcNAc:betaGal beta-1,3-N-acetylglucosaminyltransferase 5	IMAGE:487722
117977 || CXCL10 || chemokine (C-X-C motif) ligand 10	IMAGE:1493160
308875 || ATP1B3 || ATPase, Na+/K+ transporting, beta 3 polypeptide	IMAGE:136722
114193 || AFAP || actin filament associated protein	IMAGE:1084673
119785 || SDC1 || syndecan 1	IMAGE:525926
118458 || FTHFSDC1 || formyltetrahydrofolate synthetase domain containing 1	IMAGE:767068
105847 || || Homo sapiens ubiquitin-conjugating enzyme E2E 3 (UBC4/5 homolog, yeast), mRNA (cDNA clone IM/	IMAGE:795809
316641 || KPNA2 || karyopherin alpha 2 (RAG cohort 1, importin alpha 1)	IMAGE:824962
307638 || ANLN || anillin, actin binding protein (scraps homolog, Drosophila)	IMAGE:128711
115648 || ARL6IP2 || ADP-ribosylation-like factor 6 interacting protein 2	IMAGE:220395
98982 || STK6 || serine/threonine kinase 6	IMAGE:129865
118920 || PROM1 || prominin 1	IMAGE:27544
223279 || TRPV4 || transient receptor potential cation channel, subfamily V, member 4	IMAGE:379598
115701 || WARS || tryptophanyl-tRNA synthetase	IMAGE:855786
101019 || CDC42EP1 || CDC42 effector protein (Rho GTPase binding) 1	IMAGE:159462
310126 || EIF2C2 || eukaryotic translation initiation factor 2C, 2	IMAGE:110568
105624 || LY6E || lymphocyte antigen 6 complex, locus E	IMAGE:1470048
222483 || SLC2A1 || solute carrier family 2 (facilitated glucose transporter), member 1	IMAGE:25389
120627 || RNF24 || ring finger protein 24	IMAGE:47169
245567 || ||	IMAGE:132711
102564 || RTP801 || HIF-1 responsive RTP801	IMAGE:813645
109841 || PCDH7 || BH-protocadherin (brain-heart)	IMAGE:753028
330735 || UGT8 || UDP glycosyltransferase 8 (UDP-galactose ceramide galactosyltransferase)	IMAGE:2470840
312550 || RAD54B || RAD54B homolog	IMAGE:1586472
100489 || ||	IMAGE:769857
105705 || LRP8 || low density lipoprotein receptor-related protein 8, apolipoprotein e receptor	IMAGE:415554
109648 || NFIB || nuclear factor I/B	IMAGE:416959
113746 || TFAP2C || transcription factor AP-2 gamma (activating enhancer binding protein 2 gamma)	IMAGE:725680
318862 || LMO4 || LIM domain only 4	IMAGE:1706027
330614 || RASAL1 || RAS protein activator like 1 (GAP1 like)	IMAGE:2365853
223034 || KIAA1228 || KIAA1228 protein	IMAGE:824201
109924 || GPRC5B || G protein-coupled receptor, family C, group 5, member B	IMAGE:321580
105351 || PRKY || protein kinase, Y-linked	IMAGE:310034

98765 || DLX5 || distal-less homeo box 5 IMAGE:299600
222979 || ZBED4 || zinc finger, BED domain containing 4 IMAGE:827165
119100 || CCNA2 || cyclin A2 IMAGE:950690
311090 || KIAA1833 || hypothetical protein KIAA1833 IMAGE:1877561
331271 || IRAK1 || interleukin-1 receptor-associated kinase 1 IMAGE:2566158
225703 || MGC50844 || hypothetical protein MGC50844 IMAGE:281597
99275 || IRAK1 || interleukin-1 receptor-associated kinase 1 IMAGE:379200
223440 || MYO10 || myosin X IMAGE:460646
309074 || GZMB || granzyme B (granzyme 2, cytotoxic T-lymphocyte-associated serine esterase 1) IMAGE:1757321
309699 || EGFR || epidermal growth factor receptor (erythroblastic leukemia viral (v-erb-b) oncogene homolog, avian) IMAGE:241197
109366 || CDC25B || cell division cycle 25B IMAGE:786067
113314 || NFIB || nuclear factor I/B IMAGE:813265
310030 || RISC || likely homolog of rat and mouse retinoid-inducible serine carboxypeptidase IMAGE:1519143
310361 || DAPK1 || death-associated protein kinase 1 IMAGE:2043415
111855 || KIAA1357 || KIAA1357 protein IMAGE:415766
225684 || NFIB || nuclear factor I/B IMAGE:868078
117608 || GABRP || gamma-aminobutyric acid (GABA) A receptor, pi IMAGE:563598
110565 || || Homo sapiens hypothetical LOC150371 (LOC150371), mRNA IMAGE:155532
107474 || ZBED4 || zinc finger, BED domain containing 4 IMAGE:282561
330591 || SLC2A1 || solute carrier family 2 (facilitated glucose transporter), member 1 IMAGE:2547341
102224 || DAPK1 || death-associated protein kinase 1 IMAGE:364934
112578 || PARD3 || par-3 partitioning defective 3 homolog (C. elegans) IMAGE:127099
330535 || NEK2 || NIMA (never in mitosis gene a)-related kinase 2 IMAGE:2301826
115306 || || Homo sapiens transcribed sequence with weak similarity to protein sp:P39190 (H.sapiens) ALU3_HUM IMAGE:489931
309810 || || Homo sapiens clone P2-32 anti-oxidized LDL immunoglobulin light chain Fab mRNA, partial cds IMAGE:1909455
308662 || PRKX || protein kinase, X-linked IMAGE:1869486
117053 || || Homo sapiens clone ASPBLL54 immunoglobulin lambda light chain VJ region mRNA, partial cds IMAGE:236124
108180 || ISG20 || interferon stimulated gene 20kDa IMAGE:491751
311256 || DHCR7 || 7-dehydrocholesterol reductase IMAGE:2310272
222257 || GPR51 || G protein-coupled receptor 51 IMAGE:878097
116165 || || Homo sapiens cDNA FLJ26905 fis, clone RCT01427, highly similar to Ig lambda chain C regions IMAGE:66560
224925 || GYLTL1B || glycosyltransferase-like 1B IMAGE:703810
104172 || FOXC1 || forkhead box C1 IMAGE:253733
100532 || TNFSF10 || tumor necrosis factor (ligand) superfamily, member 10 IMAGE:183811
111258 || LAD1 || ladinin 1 IMAGE:121551
104346 || PLCB4 || phospholipase C, beta 4 IMAGE:1521297
223403 || PERP || PERP, TP53 apoptosis effector IMAGE:378365
225963 || KIAA1750 || KIAA1750 protein IMAGE:745011
226173 || SGCD || sarcoglycan, delta (35kDa dystrophin-associated glycoprotein) IMAGE:469898
100104 || KLK10 || kallikrein 10 IMAGE:810960
114105 || IGLL1 || immunoglobulin lambda-like polypeptide 1 IMAGE:344134
107949 || GALNT3 || UDP-N-acetyl-alpha-D-galactosamine:polypeptide N-acetylgalactosaminyltransferase 3 (GalN IMAGE:148225
102603 || MAD2L1 || MAD2 mitotic arrest deficient-like 1 (yeast) IMAGE:814701
102357 || MID1 || midline 1 (Opitz/BBB syndrome) IMAGE:897865
161326 || TYRO3 || TYRO3 protein tyrosine kinase IMAGE:343821
108202 || SFRS1 || splicing factor, arginine/serine-rich 1 (splicing factor 2, alternate splicing factor) IMAGE:813310
102645 || SLC2A1 || solute carrier family 2 (facilitated glucose transporter), member 1 IMAGE:207358
105534 || NEK2 || NIMA (never in mitosis gene a)-related kinase 2 IMAGE:462926
113361 || NFIB || nuclear factor I/B IMAGE:245860
99387 || HN1 || hematological and neurological expressed 1 IMAGE:795803
108541 || NSE1 || NSE1 IMAGE:838668

115237 || KLK6 || kallikrein 6 (neurosin, zyme)	IMAGE:809784
223615 || PRKX || protein kinase, X-linked	IMAGE:1032170
314209 || NDRG1 || N-myc downstream regulated gene 1	IMAGE:1917716
101882 || VGLL1 || vestigial like 1 (Drosophila)	IMAGE:460666
117599 || || Homo sapiens cDNA FLJ26252 fis, clone DMC03335	IMAGE:813286
314293 || GALNT3 || UDP-N-acetyl-alpha-D-galactosamine:polypeptide N-acetylgalactosaminyltransferase 3 (GalN	IMAGE:328542
330761 || LBR || lamin B receptor	IMAGE:2544309
116416 || || **Homo sapiens cDNA clone MGC:62026 IMAGE:6450688, complete cds	IMAGE:154809
309065 || || Homo sapiens transcribed sequence with weak similarity to protein ref:NP_060312.1 (H.sapiens) hypo	IMAGE:51763
106141 || RDH10 || retinol dehydrogenase 10 (all-trans)	IMAGE:1031919
101119 || KCNK1 || potassium channel, subfamily K, member 1	IMAGE:288896
114028 || MYC || v-myc myelocytomatosis viral oncogene homolog (avian)	IMAGE:812965
221131 || NFIB || nuclear factor I/B	IMAGE:855244
223111 || KRT6B || keratin 6B	IMAGE:1486118
102349 || TGFA || transforming growth factor, alpha	IMAGE:325822
330579 || BMP7 || bone morphogenetic protein 7 (osteogenic protein 1)	IMAGE:2440877
105113 || OL-64 || visceral adipose-specific SERPIN	IMAGE:813414
317229 || KIAA1579 || hypothetical protein FLJ10770	IMAGE:1658257
113465 || PPP1R14C || protein phosphatase 1, regulatory (inhibitor) subunit 14C	IMAGE:32516
106773 || || Homo sapiens cDNA clone MGC:62026 IMAGE:6450688, complete cds	IMAGE:80796
108507 || YWHAZ || tyrosine 3-monooxygenase/tryptophan 5-monooxygenase activation protein, zeta polypeptide	IMAGE:811166
115107 || ATP2B4 || ATPase, Ca++ transporting, plasma membrane 4	IMAGE:502326
220876 || TGFA || transforming growth factor, alpha	IMAGE:1553998
118594 || P2RY6 || pyrimidinergic receptor P2Y, G-protein coupled, 6	IMAGE:452588
307803 || MDA5 || melanoma differentiation associated protein-5	IMAGE:1520357
118457 || TAP1 || transporter 1, ATP-binding cassette, sub-family B (MDR/TAP)	IMAGE:841340
331261 || GPR51 || G protein-coupled receptor 51	IMAGE:2516756
100368 || GBP1 || guanylate binding protein 1, interferon-inducible, 67kDa	IMAGE:841008
101953 || MYC || v-myc myelocytomatosis viral oncogene homolog (avian)	IMAGE:417226
108089 || RPL26L1 || **ribosomal protein L26-like 1	IMAGE:358673
118642 || KHDRBS3 || KH domain containing, RNA binding, signal transduction associated 3	IMAGE:813151
102632 || ACY1L2 || aminoacylase 1-like 2	IMAGE:137139
104297 || FLJ10901 || hypothetical protein FLJ10901	IMAGE:594684
105262 || TP53BP2 || tumor protein p53 binding protein, 2	IMAGE:212198
311144 || || Homo sapiens similar to nanos (LOC375939), mRNA	IMAGE:1897178
116724 || MDA5 || melanoma differentiation associated protein-5	IMAGE:158184
115463 || SERPINE2 || serine (or cysteine) proteinase inhibitor, clade E (nexin, plasminogen activator inhibitor type	IMAGE:246722
109528 || RARRES1 || retinoic acid receptor responder (tazarotene induced) 1	IMAGE:309583
307267 || IL12RB2 || interleukin 12 receptor, beta 2	IMAGE:1587390
120223 || FOXC1 || forkhead box C1	IMAGE:358885
226855 || RGMA || repulsive guidance molecule A	IMAGE:743829
220621 || HUCEP11 || cerebral protein 11	IMAGE:454632
99435 || EGFR || epidermal growth factor receptor (erythroblastic leukemia viral (v-erb-b) oncogene homolog, avian)	IMAGE:324861
99955 || DKFZp434C0631 || hypothetical protein DKFZp434C0631	IMAGE:950355
224041 || || Homo sapiens mRNA; cDNA DKFZp761D1624 (from clone DKFZp761D1624)	IMAGE:460618
114642 || EGFR || epidermal growth factor receptor (erythroblastic leukemia viral (v-erb-b) oncogene homolog, avian)	IMAGE:669485
107390 || YWHAZ || tyrosine 3-monooxygenase/tryptophan 5-monooxygenase activation protein, zeta polypeptide	IMAGE:1031744
330846 || KRT5 || keratin 5 (epidermolysis bullosa simplex, Dowling-Meara/Kobner/Weber-Cockayne types)	IMAGE:2578894
239268 || RUNX3 || runt-related transcription factor 3	IMAGE:358842
120801 || || Homo sapiens transcribed sequence with strong similarity to protein pir:A25074 (H.sapiens) A25074 vi	IMAGE:49532
111191 || SPOCK2 || sparc/osteonectin, cwcv and kazal-like domains proteoglycan (testican) 2	IMAGE:726678

104251 || CDK7 || cyclin-dependent kinase 7 (MO15 homolog, Xenopus laevis, cdk-activating kinase)	IMAGE:470621
114892 || ART3 || ADP-ribosyltransferase 3	IMAGE:1468263
104849 || GGH || gamma-glutamyl hydrolase (conjugase, folylpolygammaglutamyl hydrolase)	IMAGE:809588
100448 || REG4 || **regenerating islet-derived family, member 4	IMAGE:951449
118765 || EPB41L4B || erythrocyte membrane protein band 4.1 like 4B	IMAGE:210698
105509 || LOC129607 || hypothetical protein LOC129607	IMAGE:207838
318210 || || Homo sapiens transcribed sequence with strong similarity to protein sp:O60269 (H.sapiens) Y514_HU	IMAGE:1710068
99938 || PHC2 || polyhomeotic-like 2 (Drosophila)	IMAGE:898328
101469 || DKK1 || dickkopf homolog 1 (Xenopus laevis)	IMAGE:669375
110771 || cig5 || viperin	IMAGE:120600
105919 || SAMD6 || sterile alpha motif domain containing 6	IMAGE:744632
102719 || UGT8 || UDP glycosyltransferase 8 (UDP-galactose ceramide galactosyltransferase)	IMAGE:254328
105839 || RUNX3 || runt-related transcription factor 3	IMAGE:291478
107602 || TRIM29 || tripartite motif-containing 29	IMAGE:377275

135 Negative Significant Genes

Gene Name	Gene ID
120339 || IRS1 || insulin receptor substrate 1	IMAGE:796284
98838 || ESR1 || estrogen receptor 1	IMAGE:725321
115462 || DNALI1 || dynein, axonemal, light intermediate polypeptide 1	IMAGE:782688
111103 || NAT1 || N-acetyltransferase 1 (arylamine N-acetyltransferase)	IMAGE:66599
330362 || FLJ20151 || hypothetical protein FLJ20151	IMAGE:2494168
110751 || TENC1 || tensin like C1 domain-containing phosphatase	IMAGE:813603
105917 || || Human clone 23948 mRNA sequence	IMAGE:49567
314918 || KIAA0882 || KIAA0882 protein	IMAGE:2284924
103054 || FLJ13710 || hypothetical protein FLJ13710	IMAGE:490965
114369 || DACH || dachshund homolog (Drosophila)	IMAGE:52021
314597 || || Homo sapiens transcribed sequences	IMAGE:1558233
107403 || QDPR || quinoid dihydropteridine reductase	IMAGE:23776
117657 || BC008967 || hypothetical gene BC008967	IMAGE:782497
221236 || || **Homo sapiens LOC375816 (LOC375816), mRNA	IMAGE:470227
220332 || DKFZp434L142 || hypothetical protein DKFZp434L142	IMAGE:823655
115623 || HSA250839 || gene for serine/threonine protein kinase	IMAGE:344959
115160 || SCUBE2 || signal peptide, CUB domain, EGF-like 2	IMAGE:1132490
112813 || ITPR1 || inositol 1,4,5-triphosphate receptor, type 1	IMAGE:471725
108855 || FLJ20151 || **hypothetical protein FLJ20151	IMAGE:594633
309686 || ||	IMAGE:683569
116862 || MAPT || microtubule-associated protein tau	IMAGE:647397
111780 || BCMP11 || breast cancer membrane protein 11	IMAGE:841621
109111 || BCMP11 || breast cancer membrane protein 11	IMAGE:119982
117547 || RAI2 || retinoic acid induced 2	IMAGE:501868
220895 || FLJ11155 || hypothetical protein FLJ11155	IMAGE:288770
178406 || GATA3 || GATA binding protein 3	IMAGE:365681
110271 || LPXN || leupaxin	IMAGE:325128
224276 || MLPH || melanophilin	IMAGE:1558642
118673 || || Homo sapiens transcribed sequences	IMAGE:782972
220479 || ||	IMAGE:399463
319994 || MAGED2 || melanoma antigen, family D, 2	IMAGE:2316397
330354 || TFF1 || trefoil factor 1 (breast cancer, estrogen-inducible sequence expressed in)	IMAGE:2504927
99416 || || Homo sapiens transcribed sequences	IMAGE:773443
226434 || || Homo sapiens cDNA FLJ12924 fis, clone NT2RP2004709.	IMAGE:279655

109952 || FLJ10724 || melanoma antigen recognized by T cells 2	IMAGE:359855
222802 || || Homo sapiens transcribed sequences	IMAGE:431988
107393 || INPP4B || inositol polyphosphate-4-phosphatase, type II, 105kDa	IMAGE:165857
222583 || SYTL2 || synaptotagmin-like 2	IMAGE:826194
224553 || CHAD || chondroadherin	IMAGE:1507713
225396 || || Homo sapiens cDNA FLJ26764 fis, clone PRS02668	IMAGE:289505
104494 || || Homo sapiens cDNA FLJ41270 fis, clone BRAMY2036387	IMAGE:153617
116130 || IRS1 || insulin receptor substrate 1	IMAGE:322005
106777 || ATP7B || ATPase, Cu++ transporting, beta polypeptide (Wilson disease)	IMAGE:266312
120064 || FOXA1 || forkhead box A1	IMAGE:954694
109865 || GPA33 || glycoprotein A33 (transmembrane)	IMAGE:1144652
105196 || SLC7A2 || solute carrier family 7 (cationic amino acid transporter, y+ system), member 2	IMAGE:132140
107435 || KIF12 || kinesin family member 12	IMAGE:214205
101265 || || Homo sapiens mRNA; cDNA DKFZp686A0815 (from clone DKFZp686A0815)	IMAGE:504959
98909 || ADCY1 || adenylate cyclase 1 (brain)	IMAGE:42389
309894 || LASP1 || LIM and SH3 protein 1	IMAGE:1631663
433331 || || *mitoch. cont. cytochrome P450, subfamily IIB (phenobarbital-inducible), polypeptide 6	IMAGE:182295
117581 || FOXA1 || forkhead box A1	IMAGE:1211831
116272 || CYBRD1 || cytochrome b reductase 1	IMAGE:838446
120709 || LAF4 || lymphoid nuclear protein related to AF4	IMAGE:51185
118640 || DACH || dachshund homolog (Drosophila)	IMAGE:29967
226202 || FLJ11280 || **hypothetical protein FLJ11280	IMAGE:239568
111946 || CACNA2D2 || calcium channel, voltage-dependent, alpha 2/delta subunit 2	IMAGE:284160
101948 || INPP4B || **inositol polyphosphate-4-phosphatase, type II, 105kDa	IMAGE:813513
221455 || NOSTRIN || nitric oxide synthase trafficker	IMAGE:854763
310822 || IL6ST || interleukin 6 signal transducer (gp130, oncostatin M receptor)	IMAGE:2018581
226316 || PREX1 || phosphatidylinositol 3,4,5-trisphosphate-dependent RAC exchanger 1	IMAGE:825270
330779 || RABEP1 || rabaptin, RAB GTPase binding effector protein 1	IMAGE:2545220
98862 || ELOVL2 || elongation of very long chain fatty acids (FEN1/Elo2, SUR4/Elo3, yeast)-like 2	IMAGE:44387
330909 || RGS5 || regulator of G-protein signalling 5	IMAGE:2569701
119997 || FMO5 || flavin containing monooxygenase 5	IMAGE:197525
220269 || ||	IMAGE:279837
110212 || NAT1 || N-acetyltransferase 1 (arylamine N-acetyltransferase)	IMAGE:195525
118440 || MGC24047 || hypothetical protein MGC24047	IMAGE:155072
115654 || SCUBE2 || signal peptide, CUB domain, EGF-like 2	IMAGE:916834
102229 || || Homo sapiens transcribed sequence with weak similarity to protein ref:NP_060312.1 (H.sapiens) hypo	IMAGE:1056516
223683 || MAN2C1 || **mannosidase, alpha, class 2C, member 1	IMAGE:415610
101559 || || Homo sapiens transcribed sequences	IMAGE:79807
310254 || RAMP2 || receptor (calcitonin) activity modifying protein 2	IMAGE:379604
103753 || RABEP1 || rabaptin, RAB GTPase binding effector protein 1	IMAGE:772890
110792 || C14orf45 || chromosome 14 open reading frame 45	IMAGE:795744
315513 || SCUBE2 || signal peptide, CUB domain, EGF-like 2	IMAGE:1691237
330411 || C4A || complement component 4A	IMAGE:2559389
307599 || HOXB4 || homeo box B4	IMAGE:1534155
113997 || DCAMKL1 || doublecortin and CaM kinase-like 1	IMAGE:277423
102455 || ASAH1 || N-acylsphingosine amidohydrolase (acid ceramidase) 1	IMAGE:855487
105889 || || Homo sapiens transcribed sequence with weak similarity to protein pir:A35363 (H.sapiens) A35363 sy	IMAGE:124315
116003 || GREB1 || GREB1 protein	IMAGE:299332
101009 || SCGB2A2 || secretoglobin, family 2A, member 2	IMAGE:964748
108183 || ||	IMAGE:1191183
99032 || ||	IMAGE:84786

330499 || LOH11CR2A || loss of heterozygosity, 11, chromosomal region 2, gene A	IMAGE:2502722
223489 || CLIC6 || chloride intracellular channel 6	IMAGE:854831
114720 || LRP2 || low density lipoprotein-related protein 2	IMAGE:143846
220510 || MGC18216 || hypothetical protein MGC18216	IMAGE:428184
223020 || FLJ23153 || likely ortholog of mouse tumor necrosis-alpha-induced adipose-related protein	IMAGE:80050
108584 || FLJ11280 || **hypothetical protein FLJ11280	IMAGE:245341
98724 || || Homo sapiens cDNA FLJ26212 fis, clone ADG07859	IMAGE:204684
100769 || LRP2 || low density lipoprotein-related protein 2	IMAGE:183773
112348 || ACADSB || acyl-Coenzyme A dehydrogenase, short/branched chain	IMAGE:243100
99429 || || Homo sapiens transcribed sequences	IMAGE:358314
221066 || || Homo sapiens transcribed sequences	IMAGE:266631
226045 || LOC90557 || hypothetical protein BC016861	IMAGE:590310
222446 || PACE4 || paired basic amino acid cleaving system 4	IMAGE:435718
101636 || SLC39A6 || solute carrier family 39 (zinc transporter), member 6	IMAGE:52933
103482 || PKIB || protein kinase (cAMP-dependent, catalytic) inhibitor beta	IMAGE:152289
224616 || ABCC8 || ATP-binding cassette, sub-family C (CFTR/MRP), member 8	IMAGE:1558108
108925 || FLJ25429 || hypothetical protein FLJ25429	IMAGE:124239
331200 || LRP2 || low density lipoprotein-related protein 2	IMAGE:2464693
100957 || RGS5 || regulator of G-protein signalling 5	IMAGE:853809
117713 || || Homo sapiens cDNA FLJ12935 fis, clone NT2RP2004982.	IMAGE:32962
309209 || || Homo sapiens transcribed sequence with strong similarity to protein ref:NP_079038.1 (H.sapiens) hyp	IMAGE:1632235
246144 || ||	IMAGE:26883
118812 || MGC18216 || hypothetical protein MGC18216	IMAGE:220069
225175 || || Homo sapiens cDNA FLJ42585 fis, clone BRACE3009237	IMAGE:845037
114914 || PLAT || plasminogen activator, tissue	IMAGE:813841
101421 || || Homo sapiens mRNA full length insert cDNA clone EUROIMAGE 980547	IMAGE:232860
311752 || STATIP1 || signal transducer and activator of transcription 3 interacting protein 1	IMAGE:1686600
114743 || GLRB || glycine receptor, beta	IMAGE:810618
116038 || IL6ST || interleukin 6 signal transducer (gp130, oncostatin M receptor)	IMAGE:753743
330278 || IGF1R || insulin-like growth factor 1 receptor	IMAGE:2488804
119736 || LOC222171 || hypothetical protein LOC222171	IMAGE:924741
116219 || GATA3 || GATA binding protein 3	IMAGE:214068
100840 || C6orf211 || chromosome 6 open reading frame 211	IMAGE:785795
101511 || ACOX2 || acyl-Coenzyme A oxidase 2, branched chain	IMAGE:85450
221470 || ACACB || acetyl-Coenzyme A carboxylase beta	IMAGE:1635307
319669 || || Homo sapiens cDNA: FLJ22066 fis, clone HEP10611	IMAGE:207274
98556 || SLC1A2 || solute carrier family 1 (glial high affinity glutamate transporter), member 2	IMAGE:52990
103089 || || Homo sapiens transcribed sequences	IMAGE:1557637
118262 || || **Homo sapiens transcribed sequence with strong similarity to protein ref:NP_079038.1 (H.sapiens) hy	IMAGE:66400
117245 || HPN || hepsin (transmembrane protease, serine 1)	IMAGE:208413
311521 || SEC14L2 || SEC14-like 2 (S. cerevisiae)	IMAGE:2019750
109558 || NPY1R || neuropeptide Y receptor Y1	IMAGE:33045
313631 || TSPAN-1 || tetraspan 1	IMAGE:2320932
119404 || SOX17 || SRY (sex determining region Y)-box 17	IMAGE:742062
106610 || DACH || dachshund homolog (Drosophila)	IMAGE:132326
106139 || GSTM2 || glutathione S-transferase M2 (muscle)	IMAGE:713922
308499 || CHN2 || chimerin (chimaerin) 2	IMAGE:1526036
116211 || ELN || elastin (supravalvular aortic stenosis, Williams-Beuren syndrome)	IMAGE:810934
101842 || APXL2 || apical protein 2	IMAGE:281681
239150 || PACE4 || **paired basic amino acid cleaving system 4	IMAGE:415962
