# Supplementary material for: TP53 mutation status and gene expression profiles are powerful prognostic markers of breast cancer
Source: Breast Cancer Res. 2007 May 15;9(3):R30. doi: 10.1186/bcr1675 (PMC1929092; doi:10.1186/bcr1675)
Supplement: Additional file 2 — A table listing TP53 mutations. [file bcr1675-S2.pdf]

Table S2: TP53 mutations

Patient identity	Exon	Codon	Codon change	Base change	Amino acid change	Comments
ULL - 007	6	195	ATC > ACC	T > C	ILE > THR	
ULL - 009	8	273	CGT > TGT	C > T	ARG > CYS	
ULL - 023	Intron 6			A > G		Intron 6 (upstream exon 7)
ULL - 038	5	138	GCC > GTC	C > T	ALA > VAL	Additional mutation in intron 7
ULL - 051	5	138	GCC > GTC	C > T	ALA > VAL	
ULL - 055	8	273	CGT > TGT	C > T	ARG > CYS	
ULL - 056	7	249	AGG > ATG	G > T	ARG > MET	
ULL - 057	5	163	TAC > TGC	A > G	TYR > CYS	
ULL - 065	8	273	CGT > TGT	C > T	ARG > CYS	
ULL - 069	6	193	CAT > CTT	A > T	HIS > LEU	
ULL - 071	8	273	CGT > GGT	C > G	ARG > GLY	
ULL - 080	5	143	GTG > ATG	G > A	VAL > MET	
ULL - 083	7	259	GAC > TAC	G > T	ASP > TYR	
ULL - 090	Intron 6			G > T		Intron 6 (downstream exon 6)
ULL - 094	7	245	GGC > AGC	G > A	GLY > SER	
ULL - 096	5		18 bp insertion			
ULL - 098	6	218	GTG > ATG	G > A	VAL > MET	
ULL - 099	5	173	GTG > CTG	G > C	VAL > LEU	
ULL - 101	8	273	CGT > TGT	C > T	ARG > CYS	
ULL - 106	5	159	GCC > GAC	C > A	ALA > ASP	
ULL - 113	4	113	TTC > GTC	T > G	PHE > VAL	
ULL - 125	8	273	CGT > CTT	G > T	ARG > LEU	
ULL - 131	10	342	CGA > TGA	C > T	ARG > STOP	
ULL - 139	8	286	7 bp deletion			
ULL - 142	11	393	4 bp deletion			
ULL - 155	5	175	CGC > CAC	G > A	ARG > HIS	
ULL - 167	7	251	ATC > AGC	T > G	ILE > SER	
ULL - 171	5	175	CGC > CAC	G > A	ARG > HIS	
ULL - 173	7	248	CGG > TGG	C > T	ARG > TRP	
ULL - 177	4	110	CGT > CCT	G > C	ARG > PRO	
ULL - 179	6	196	22 bp deletion			
ULL - 188	6	216	GTG > ATG	G > A	VAL > MET	
ULL - 198	5	179	GTG > CTG	G > C	VAL > LEU	
ULL - 202	5	163	TAC > CAC	T > C	TYR > HIS	
ULL - 209	8	282	CGG > TGG	C > T	ARG > TRP	
ULL - 212	5	126	TAC > GAC	T > G	TYR > ASP	
ULL - 215	8	278	CCT > CTT	C > T	PRO > LEU	
ULL - 221	7	239	AAC > AGC	A > G	ASN > SER	
ULL - 226	6	220	TAT > TGT	A > G	TYR > CYS	
ULL - 238	7	244	GGC > GAC	G > A	GLY > ASP	
ULL - 243	5	175	CGC > CAC	G > A	ARG > HIS	
ULL - 244	4	38	CAA > TAA	C > T	GLN > STOP	
ULL - 246	7	241	TCC > TTC	C > T	SER > PHE	
ULL - 250	7	248	CGG > CAG	G > A	ARG > GLN	
ULL - 263	4	89	11 bp insertion			
ULL - 267	9	321	1 bp deletion	del A		
ULL - 271	3	29	1 bp insertion	ins A		
ULL - 274	8	273	CGT > CAT	G > A	ARG > HIS	
