# Supplementary figures and images for: TP53 mutation status and gene expression profiles are powerful prognostic markers of breast cancer
Source: Breast Cancer Res. 2007 May 15;9(3):R30. doi: 10.1186/bcr1675 (PMC1929092; doi:10.1186/bcr1675)

Supplementary Figure S1

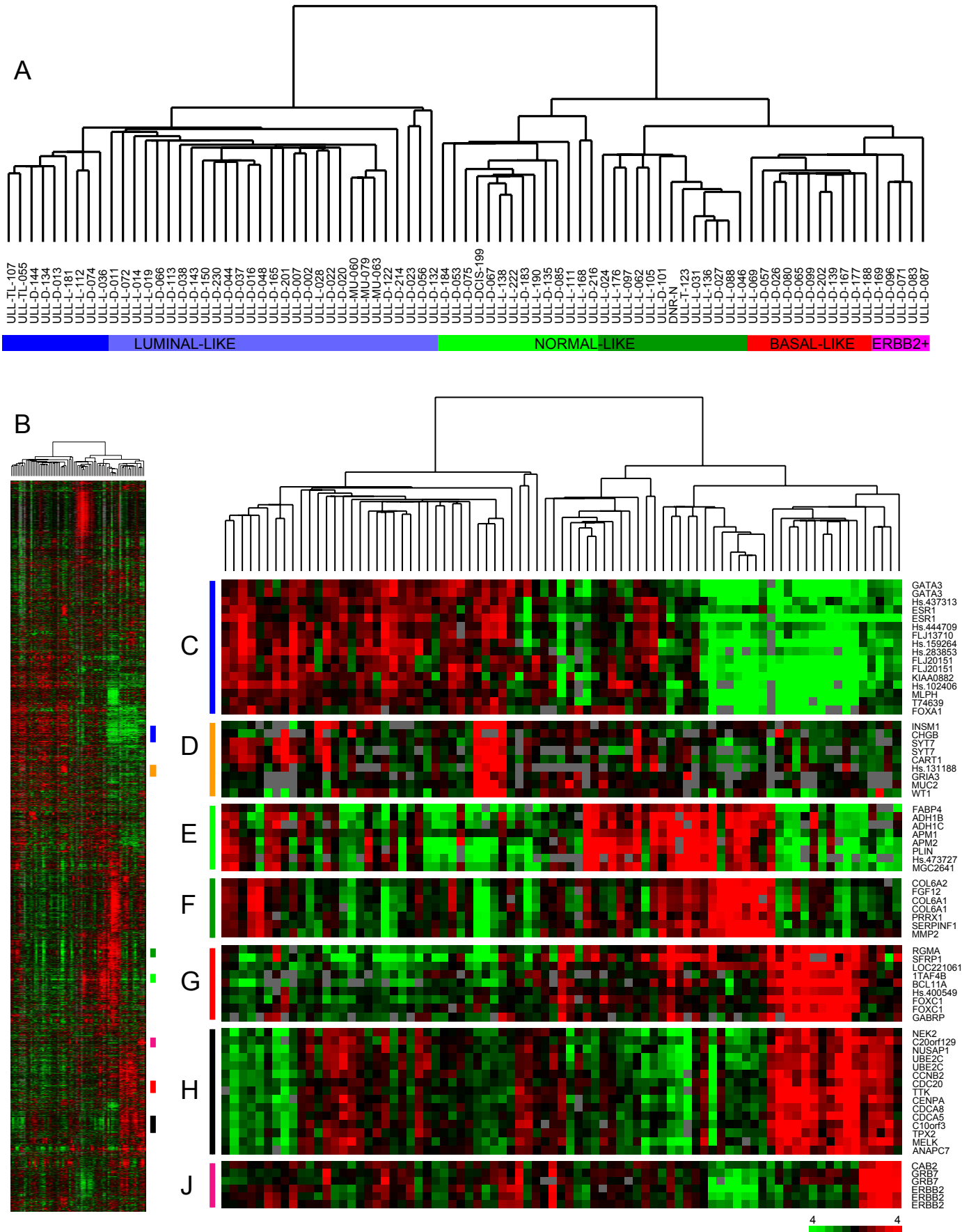

Supplement: Additional file 4 — A figure showing the hierarchical clustering using the total set of genes. [file bcr1675-S4.pdf]
